# Association of Prescription Co-payment With Adherence to Glucagon-Like Peptide-1 Receptor Agonist and Sodium-Glucose Cotransporter-2 Inhibitor Therapies in Patients With Heart Failure and Diabetes

**DOI:** 10.1001/jamanetworkopen.2023.16290

**Published:** 2023-06-01

**Authors:** Utibe R. Essien, Balvindar Singh, Gretchen Swabe, Amber E. Johnson, Lauren A. Eberly, Rishi K. Wadhera, Khadijah Breathett, Muthiah Vaduganathan, Jared W. Magnani

**Affiliations:** 1 Division of General Internal Medicine and Health Services Research, David Geffen School of Medicine, University of California, Los Angeles; 2 Center for the Study of Healthcare Innovation, Implementation and Policy, VA Greater Los Angeles Healthcare System, Los Angeles, California; 3 Department of Medicine, University of Pittsburgh School of Medicine, Pittsburgh, Pennsylvania; 4 Division of Cardiovascular Medicine, Department of Medicine, Hospital of the University of Pennsylvania, Philadelphia; 5 Penn Cardiovascular Outcomes, Quality, and Evaluative Research Center, Cardiovascular Institute, University of Pennsylvania, Philadelphia; 6 Penn Cardiovascular Center for Health Equity and Social Justice, University of Pennsylvania, Philadelphia; 7 Richard A. and Susan F. Smith Center for Outcomes Research in Cardiology, Beth Israel Deaconess Medical Center, Boston, Massachusetts; 8 Division of Cardiovascular Medicine, Indiana University, Indianapolis; 9 Division of Cardiovascular Medicine, Brigham and Women’s Hospital and Harvard Medical School, Boston, Massachusetts; 10 Center for Research on Health Care, University of Pittsburgh Department of Medicine, Pittsburgh, Pennsylvania

## Abstract

**Importance:**

Type 2 diabetes (T2D) and heart failure (HF) prevalence are rising in the US. Although glucagon-like peptide-1 receptor agonists (GLP1-RA) and sodium-glucose cotransporter 2 inhibitors (SGLT2i) improve outcomes for these conditions, high out-of-pocket costs may be associated with reduced medication adherence.

**Objective:**

To compare 1-year adherence to GLP1-RA and SGLT2i therapies by prescription co-payment level in individuals with T2D and/or HF.

**Design, Setting, and Participants:**

This retrospective cohort study used deidentified data from Optum Insight’s Clinformatics Data Mart Database of enrollees with commercial and Medicare health insurance plans. Individuals aged 18 years or older with T2D and/or HF who had a prescription claim for a GLP1-RA or SLGT2i from January 1, 2014, to September 30, 2020, were included.

**Exposures:**

Prescription co-payment, categorized as low (<$10), medium ($10 to<$50), and high (≥$50).

**Main Outcomes and Measures:**

The primary outcome was medication adherence, defined as a proportion of days covered (PDC) of 80% or greater at 1 year. Logistic regression models were used to examine the association between co-payment and adherence, adjusting for patient demographics, medical comorbidities, and socioeconomic factors.

**Results:**

A total of 94 610 individuals (mean [SD] age, 61.8 [11.4] years; 51 226 [54.1%] male) were prescribed GLP1-RA or SGLT2i therapy. Overall, 39 149 individuals had a claim for a GLP1-RA, of whom 25 557 (65.3%) had a PDC of 80% or greater at 1 year. In fully adjusted models, individuals with a medium (adjusted odds ratio [AOR], 0.62; 95% CI, 0.58-0.67) or high (AOR, 0.47; 95% CI, 0.44-0.51) co-payment were less likely to have a PDC of 80% or greater with a GLP1-RA compared with those with a low co-payment. Overall, 51 072 individuals had a claim for an SGLT2i, of whom 37 339 (73.1%) had a PDC of 80% or greater at 1 year. Individuals with a medium (AOR, 0.67; 95% CI, 0.63-0.72) or high (AOR, 0.68; 95% CI, 0.63-0.72) co-payment were less likely to have a PDC of 80% or greater with an SGLT2i compared with those with a low co-payment.

**Conclusions and Relevance:**

In this cohort study of individuals with T2D and/or HF, 1-year adherence to GLP1-RA or SGLT2i therapies was highest among individuals with a low co-payment. Improving adherence to guideline-based therapies may require interventions that reduce out-of-pocket prescription costs.

## Key Points

**Question:**

Is prescription co-payment associated with adherence to glucagon-like peptide-1 receptor agonist (GLP1-RA) or sodium-glucose cotransporter 2 inhibitor (SGLT2i) therapies in individuals with diabetes and/or heart failure?

**Findings:**

In this cohort study of 94 610 US adults, after adjustment for clinical and socioeconomic factors such as household income, educational attainment, race, and ethnicity, individuals with higher levels of prescription co-payment were significantly less likely to achieve 1-year adherence to GLP1-RA and SGLT2i medications.

**Meaning:**

The findings suggest that improving adherence to guideline-based therapies for the prevention of adverse cardiovascular outcomes warrants policy-level interventions to reduce prescription co-payment.

## Teaser

This cohort study compares rates of 1-year adherence to glucagon-like peptide-1 receptor agonist or sodium-glucose cotransporter 2 inhibitor therapies by co-payment level among US adults with type 2 diabetes and/or heart failure.

## Introduction

Cardiometabolic disease remains the most common cause of disability and death in adults worldwide.^1,2^ The prevalence of type 2 diabetes (T2D) and heart failure (HF) are steadily rising in the US, increasing to over 37 million and 6 million individuals, respectively, as of 2021.^3,4^ While physical activity, diet monitoring, and other modes of modifiable risk factor control are critical to the treatment of T2D and HF, prevention of chronic complications also requires effective adherence to prescription medications with demonstrated potential to improve outcomes. However, evidence-based management is often costly, limiting treatment access, initiation, and adherence.^5^

Two emerging classes of evidence-based cardiovascular therapies are glucagon-like peptide-1 receptor agonists (GLP1-RA) and sodium-glucose cotransporter 2 inhibitors (SGLT2i).^6^ These medications have demonstrated significant reduction in morbidity and mortality for patients with T2D and HF^7,8^ and have been added to contemporary diabetes and cardiovascular guidelines to decrease risk of adverse events.^9^ Despite their proven benefit, prior research has shown that high out-of-pocket costs associated with GLP1-RA and SGLT2i therapies represent a significant barrier to medication initiation.^10,11,12^ Yet, less is known about how cost sharing by patients, specifically through prescription co-payment, influences long-term adherence to these medications.

In this analysis, we examined the association of prescription co-payment with adherence to GLP1-RA and SGLT2i therapies in a nationwide cohort of individuals with T2D and/or HF. We hypothesized that in a cohort of commercially insured individuals, those with lower co-payments would have increased likelihood of 12-month medication adherence, even after controlling for sociodemographic factors associated with medication adherence.

## Methods

### Data Sources

We used data from Optum Insight’s deidentified Clinformatics Data Mart Database to conduct this retrospective cohort study. Clinformatics is a large, geographically diverse, administrative claims database of over 68 million enrollees with commercial and Medicare health insurance plans.^13,14^ The database consists of annual inpatient, outpatient, emergency department, and pharmacy claims for all enrollees. Medical claims include *International Classification of Diseases, Ninth Revision, Clinical Modification (ICD-9-CM)* and *International Statistical Classification of Diseases, Tenth Revision, Clinical Modification (ICD-10-CM)* codes and site-of-service codes. The University of Pittsburgh institutional review board determined this research was exempt from regulatory requirements, as it did not constitute human participants research, and waived informed consent because the data were deidentified. We followed the Strengthening the Reporting of Observational Studies in Epidemiology (STROBE) reporting guideline for study design, execution, analysis, interpretation, and reporting.

### Cohort Eligibility Criteria

We specified that individuals in the cohort had at least 6 months of continuous enrollment in Clinformatics prior to their initial prescription fill and at least 12 months of enrollment following their initial fill date or index date. We shifted days forward with overlapping coverage, assuming an individual would finish the existing medication supply before starting the new supply. We used pharmacy claims to identify all individuals with a new prescription, defined as having no record of a GLP1-RA or SGLT2i prescription fill in the previous 6 months, from January 1, 2014, to September 30, 2020. We used the generic product identifier to capture GLP1-RA prescriptions, including albiglutide, dulaglutide, exenatide, liraglutide, lixisenatide, or semaglutide. The SGLT2i prescriptions were canagliflozin, dapagliflozin, empagliflozin, or ertugliflozin. Combination therapy was defined as individuals taking 1 agent of each medication class concurrently for the duration of follow-up.

**Figure 1.**
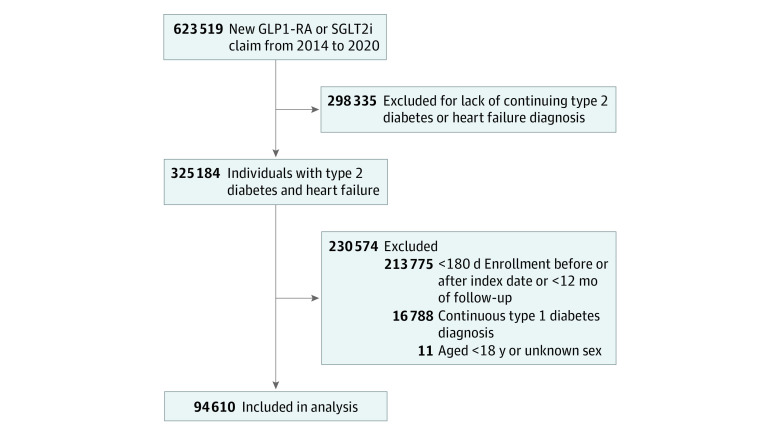
Identification of the Study Sample GLP1-RA indicates glucagon-like peptide-1 receptor agonist; SGLT2i, sodium-glucose cotransporter 2 inhibitor.

We excluded individuals who did not have a diagnosis of T2D or HF of any type, using established *ICD-9-CM* and *ICD-10-CM* diagnosis codes to identify these individuals.^15^ All administrative codes used in the analysis are provided in eTable 1 in Supplement 1. We also excluded individuals who discontinued medications prior to 12 months or did not have 12 months of available prescription information for either medication class. We excluded individuals younger than 18 years or with unknown sex, those with a diagnosis of type 1 diabetes, and those with fewer than 180 days of enrollment in Clinformatics before the initial fill date. Figure 1 shows the flow diagram for selection criteria of the final cohort used in this study.

### Study Outcomes

The primary outcome was 12-month adherence to GLP1-RA or SGLT2i therapies, defined as the proportion of days covered (PDC) for the prescription of interest. Consistent with prior studies, adequate adherence (hereafter referred to as *adherence*) was characterized by having a PDC of 80% or greater over 12 months.^16,17,18^ The phase of medication adherence assessed in this analysis was the initiation phase.^19^ To calculate PDC, we counted the number of days in which medication was available to an individual over the course of 365 days of follow-up as determined by fill dates and days supplied as listed on claims.

### Independent Variable and Covariates

The independent variable of interest was 30-day prescription co-payment. Co-payment was obtained from pharmacy claims and defined as the individual-level median over the course of 12 months.^13^ We categorized co-payment as low (<$10), medium ($10 to<$50), or high (≥$50) based on the distribution of co-payment data, with low co-payment serving as the referent. Co-payment was not adjusted for inflation. Co-payment ranges were similar for both medication types. Hence, we used the same classification system for both GLP1-RA and SGLT2i therapies.

We identified additional patient demographic variables, including age, sex (female, male), race, and ethnicity, which are collected by Clinformatics for each individual member at the time of enrollment and as previously described.^14,20^ The available race and ethnicity categories were non-Hispanic Asian, non-Hispanic Black, Hispanic (all races), and non-Hispanic White, all predefined by Clinformatics^14^; race and ethnicity were included in the analysis because of their reported association with prescription medication access and adherence.^10,11,21,22^ Patient-level socioeconomic data included (1) regional geographic location within the US, (2) median household income range (estimated and validated from surveys of US households using a comprehensive set of variables that encompass 9-digit postal code, Internal Revenue Service data, address-level home value, aggregated credit, and short-term loans), (3) educational attainment level (derived from census data at the 9-digit postal code level), (4) Medicare insurance enrollment type, and (5) presence of a medication deductible (ie, in which an individual contributed any amount toward their medication cost).^14,20^ We assessed medical comorbidities prior to the index prescription fill through the Elixhauser Comorbidity Index, which uses *ICD-9* and *ICD-10* diagnosis codes to capture a range of chronic and acute medical conditions, including cardiometabolic disease, malignant tumors, substance use disorder, and mental health conditions.^14,23^ We also quantified health care utilization, including the number of primary care physician and specialty physician (eg, cardiology and endocrinology) visits, during the follow-up period.

### Statistical Analysis

We examined age by its mean and SD and all other continuous variables by their median and IQR. Individuals without race, ethnicity, or educational level data were labeled as “unknown” for that variable, which was then treated as its own category rather than a null value.^14^ We compared sociodemographic and clinical characteristics across co-payment levels (ie, low, medium, and high) and by medication type (ie, GLP1-RA and SLGT2i).

We used multivariable logistic regression models to examine the association between co-payment category (with the lowest category of co-payment serving as the reference group) and 1-year adherence to GLP1-RA or SLGT2i therapy. Informed by a socioecological framework to assess multilevel upstream social and downstream individual factors that influence health, we used a 3-step adjustment to assess our primary outcome.^24^ Model 1 adjusted for demographic information, including age, sex, race, and ethnicity. Model 2 additionally adjusted for clinical factors using the Elixhauser Comorbidity Index score and health care utilization characteristics. Model 3 further adjusted for socioeconomic factors, including median household income, educational attainment level, regional geographic location, insurance type, and year of index prescription fill. We performed a sensitivity analysis to assess the effect modification of sociodemographic factors on the association between medication co-payment and adherence. We fit the models using a 2-way interaction term between co-payment and each sociodemographic factor (eg, sex, race, ethnicity, income, educational level, and insurance type).

For all analyses, we used a 2-tailed *P* value of <.05 to define statistical significance. Statistical analyses were performed using SAS, version 9.4 (SAS Institute Inc).

## Results

### Baseline Characteristics

The final cohort comprised 94 610 individuals who initiated GLP1-RA or SGLT2i therapy between January 1, 2014, and September 30, 2020, including 43 384 (45.9%) female individuals and 51 226 (54.1%) male individuals with an overall mean (SD) age of 61.8 (11.4) years. The cohort included 3890 (4.1%) Asian individuals, 13 553 (14.3%) Black individuals, 15 096 (16.0%) Hispanic individuals, 57 916 (61.2%) White individuals, and 3991 (4.2%) with unknown race and ethnicity. Patient demographic, clinical, and socioeconomic characteristics by medication type and co-payment level are shown in Table 1. Individuals with a low co-payment were more likely to be Black or Hispanic, reside in neighborhoods with the lowest level of median household income, and have less than a bachelor’s degree as their highest level of educational attainment compared with those with higher co-payments. The overall cohort also included 4389 individuals (4.6%) who received both a GLP1-RA and an SGLT2i medication during the study period; sociodemographic and clinical characteristics for these individuals are shown in eTable 2 in Supplement 1.

**Table 1.** Baseline Characteristics by Medication Type and Co-payment Level for Patients with Diabetes and Heart Failure^a^

Characteristic	Patients^b^					
	GLP1-RA monotherapy (n = 39 149)			SGLT2i monotherapy (n = 50 892)		
	Low co-payment (n = 11 689)	Medium co-payment (n = 11 988)	High co-payment (n = 15 472)	Low co-payment (n = 13 425)	Medium co-payment (n = 24 000)	High co-payment (n = 13 467)
Age, mean (SD), y	62.4 (11.0)	60.4 (11.6)	61.4 (11.6)	63.4 (11.0)	59.8 (11.3)	66.0 (10.5)
Sex						
Female	7356 (62.9)	6057 (50.5)	7550 (48.8)	6860 (51.1)	8598 (35.8)	5046 (37.5)
Male	4333 (37.1)	5931 (49.5)	7922 (51.2)	6565 (48.9)	15402 (64.2)	8421 (62.5)
Race and ethnicity						
Asian	289 (2.5)	297 (2.5)	388 (2.5)	748 (5.6)	1219 (5.1)	806 (6.0)
Black	2690 (23.0)	1663 (13.9)	1806 (11.7)	2603 (19.4)	2825 (11.8)	1355 (10.1)
Hispanic	2201 (18.8)	1531 (12.8)	2215 (14.3)	2779 (20.7)	3759 (15.7)	1798 (13.4)
White	5956 (51.0)	8105 (67.6)	10 410 (67.3)	6458 (48.1)	15 340 (63.9)	8989 (66.7)
Unknown	553 (4.7)	392 (3.3)	653 (4.2)	837 (6.2)	857 (3.6)	699 (5.2)
Geographic region						
East North Central	1111 (8.3)	956 (8.2)	2013 (16.8)	2211 (14.3)	1111 (8.3)	3769 (28.0)
East South Central	688 (5.1)	611 (5.2)	798 (6.7)	798 (5.2)	688 (5.1)	1351 (10.0)
Middle Atlantic	900 (6.7)	714 (6.1)	378 (3.2)	1010 (6.5)	900 (6.7)	960 (7.1)
Mountain	856 (6.4)	938 (8.0)	829 (6.9)	1512 (9.8)	856 (6.4)	1687 (12.5)
New England	844 (6.3)	525 (4.5)	210 (1.8)	418 (2.7)	844 (6.3)	419 (3.1)
Pacific	1092 (8.1)	800 (6.8)	1345 (11.2)	1382 (8.9)	1092 (8.1)	2762 (20.5)
South Atlantic	4015 (29.9)	3608 (30.9)	3595 (30.0)	3596 (23.2)	4015 (29.9)	6769 (50.3)
West North Central	952 (7.1)	979 (8.4)	1156 (9.6)	1321 (8.5)	952 (7.1)	1784 (13.2)
West South Central	2958 (22.0)	2552 (21.8)	1654 (13.8)	3208 (20.7)	2958 (22.0)	4481 (33.3)
Unknown	9 (0.1)	6 (0.1)	10 (0.1)	16 (0.1)	9 (0.1)	18 (0.1)
Median household income, $, thousands						
<40	5920 (50.7)	2963 (24.7)	3627 (23.4)	6010 (44.8)	5249 (21.9)	2909 (21.6)
40 to <50	1023 (8.8)	971 (8.1)	1273 (8.2)	1214 (9.0)	1896 (7.9)	1134 (8.4)
50 to <60	807 (6.9)	1051 (8.8)	1412 (9.1)	1029 (7.7)	2017 (8.4)	1280 (9.5)
60 to <75	832 (7.1)	1508 (12.6)	1961 (12.7)	1009 (7.5)	2791 (11.6)	1663 (12.3)
75 to <100	891 (7.6)	1999 (16.7)	2680 (17.3)	1125 (8.4)	4151 (17.3)	2491 (18.5)
≥100	1002 (8.6)	2998 (25.0)	3732 (24.1)	1664 (12.4)	6733 (28.1)	3462 (25.7)
Unknown	1214 (10.4)	498 (4.2)	787 (5.1)	1374 (10.2)	1163 (4.9)	708 (5.3)
Educational attainment level						
<12th Grade	130 (1.1)	72 (0.6)	62 (0.4)	186 (1.4)	152 (0.6)	72 (0.5)
High school diploma	5440 (46.5)	3490 (29.1)	4386 (28.4)	5891 (43.9)	6820 (28.4)	3559 (26.4)
<Bachelor degree	4909 (42.0)	6696 (55.9)	8658 (56.0)	5713 (42.6)	13 138 (54.7)	7581 (56.3)
≥Bachelor degree	759 (6.5)	1515 (12.6)	1973 (12.8)	976 (7.3)	3405 (14.2)	1996 (14.8)
Unknown	451 (3.9)	215 (1.8)	393 (2.5)	659 (4.9)	485 (2.0)	439 (3.3)
Insurance type						
Commercial	2176 (18.6)	7129 (59.5)	8053 (52.0)	3355 (25.0)	16 196 (67.5)	4171 (31.0)
Medicare Part D beneficiary	9513 (81.4)	4859 (40.5)	7419 (48.0)	10 070 (75.0)	7804 (32.5)	9296 (69.0)
Medicare, unspecified	2111 (18.1)	4676 (39.0)	7169 (46.3)	1832 (13.7)	7518 (31.3)	9076 (67.4)
Low-income subsidy	4341 (37.1)	133 (1.1)	157 (1.0)	5355 (39.9)	235 (1.0)	146 (1.1)
Dual Medicare and Medicaid	3061 (26.2)	50 (0.4)	93 (0.6)	2883 (21.5)	51 (0.2)	74 (0.5)
Time from index date to enrollment, median (IQR), mo	30.5 (23.5-41.5)	30.4 (22.8-43.1)	30.7 (23.9-41.7)	25.4 (18.2-37.8)	26.7 (18.6-39.8)	26.9 (18.9-40.2)
Medication deductible	3857 (33.0)	1869 (15.6)	4015 (26.0)	4619 (34.4)	3516 (14.7)	4152 (30.8)
Medical comorbidities						
Diabetes-qualifying diagnosis	11 513 (98.5)	11 857 (98.9)	15 346 (99.2)	13 281 (98.9)	23 813 (99.2)	13 332 (99.0)
Heart failure– qualifying diagnosis	1921 (16.4)	992 (8.3)	1175 (7.6)	1818 (13.5)	1578 (6.6)	1246 (9.3)
Elixhauser comorbidities, median (IQR), No.	7 (5-10)	6 (4-8)	5 (4-8)	6 (4-9)	5 (4-7)	5 (4-8)
Healthcare utilization encounters, median (IQR), No.						
Cardiology	0 (0-2)	0 (0-1)	0 (0-1)	0 (0-1)	0 (0-1)	0 (0-1)
Endocrinology	0 (0-0)	0 (0-0)	0 (0-0)	0 (0-0)	0 (0-0)	0 (0-0)
Primary care practitioner	21 (11-38)	15 (8-26)	12 (7-22)	16 (8-29)	11 (6-19)	12 (7-21)
Index prescription fill year						
2014	76 (0.2)	36 (0.1)	49 (0.1)	43 (0.1)	56 (0.1)	36 (0.1)
2015	106 (0.3)	102 (0.3)	82 (0.2)	76 (0.2)	122 (0.2)	77 (0.2)
2016	1309 (3.3)	2033 (5.2)	1527 (3.9)	1468 (2.9)	3533 (6.9)	1445 (2.8)
2017	2387 (6.1)	2952 (7.5)	3276 (8.4)	2295 (4.5)	5167 (10.1)	2434 (4.8)
2018	3351 (8.6)	3181 (11.4)	4471 (11.4)	2405 (4.7)	4991 (9.8)	2406 (4.7)
2019	4346 (11.1)	3613 (9.2)	5955 (15.2)	3774 (7.4)	6006 (11.8)	3785 (7.4)

Abbreviations: GLP1-RA, glucagon-like peptide-1 receptor agonist; SGLT2i, sodium-glucose cotransporter 2 inhibitor.

^a^ Low co-payment was defined as less than $10; medium, $10 to less than $50; and high, $50 or greater.

^b^ Data are presented as number (percentage) of patients unless otherwise indicated.

### Adherence to GLP1-RA Therapies by Co-payment Level

**Figure 2.**
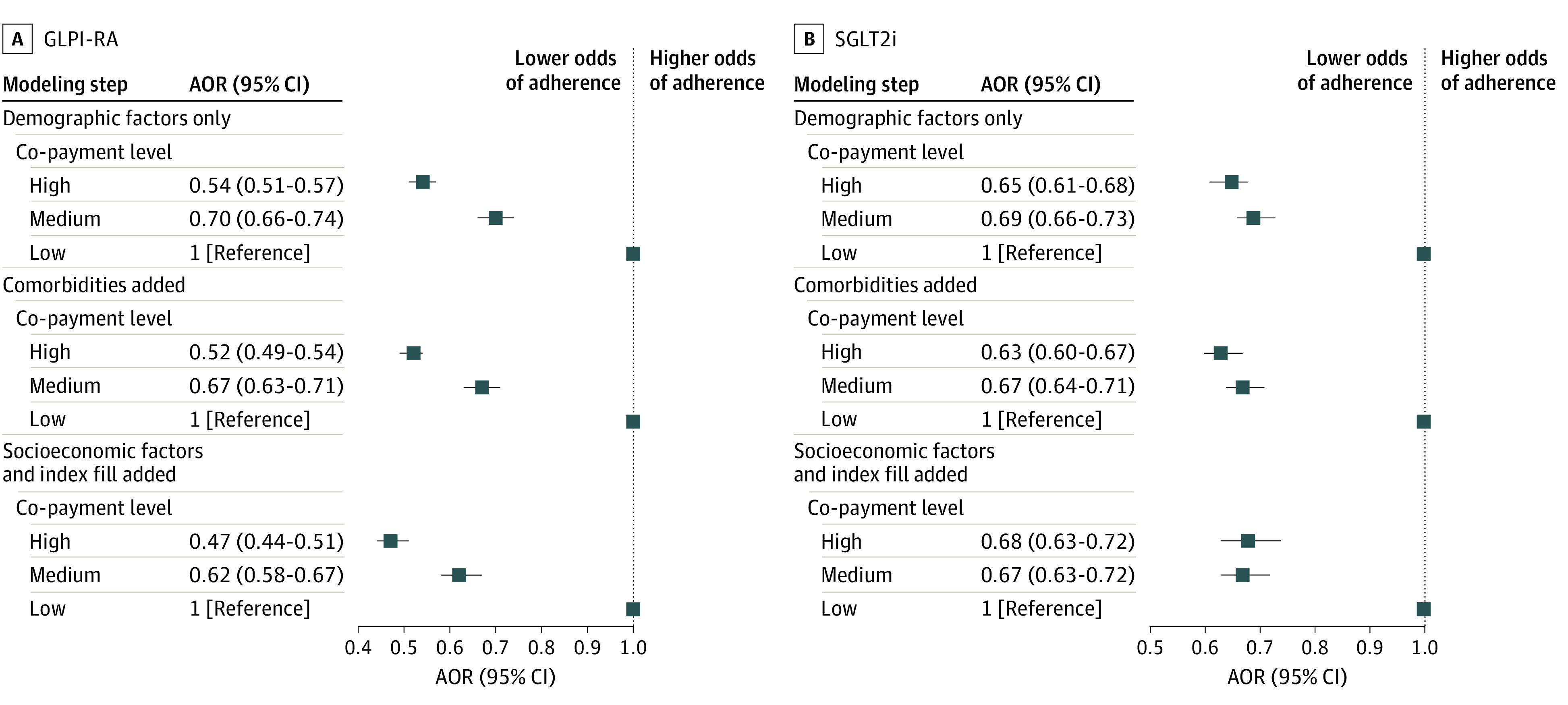
Adjusted Odds Ratios for Adherence to Glucagon-Like Peptide-1 Receptor Agonist (GLP1-RA) and Sodium-Glucose Cotransporter 2 Inhibitor (SGLT2i) Therapies by Co-payment Level The sequential logistic regression modeling approach considered patient demographics only (model 1), then added medical comorbidities (model 2) and patient socioeconomic factors and index prescription fill year (model 3). Adjusted odds ratios (AORs) of adherence compared a proportion of days covered of more than 80% across co-payment levels. Squares indicate AORs, with horizontal lines representing 95% CIs. Low co-payment was defined as less than $10; medium, $10 to less than $50; and high, $50 or greater.

The final cohort included 39 149 individuals who had a pharmacy claim for a GLP1-RA medication, including 11 689 (29.9%) with a low co-payment, 11 988 (30.6%) with a medium co-payment, and 15 472 (39.5%) with a high co-payment. Overall, 25 557 individuals (65.3%) achieved 12-month adherence, defined as having a PDC of 80% or greater, for GLP1-RA use at 1 year. Adherence varied by co-payment level, including 8407 (71.9%) with a low co-payment, 7876 (65.7%) with a medium co-payment, and 9274 (59.9%) with a high co-payment having a PDC of 80% or greater (*P* < .001) (Table 2). The odds ratio (OR) changes between modeling steps by co-payment level are shown in Figure 2A. In the fully multivariable-adjusted model, which included demographic, clinical, and socioeconomic factors and year of index prescription fill (model 3), individuals with a medium co-payment were significantly less likely to have 12-month adherence to GLP1-RA therapies compared with those with a low co-payment (adjusted OR [AOR], 0.62; 95% CI, 0.58-0.67). Individuals with a high co-payment were less likely to have 12-month adherence to GLP1-RA therapies compared with those with a low co-payment (AOR, 0.47; 95% CI, 0.44-0.51). Sensitivity analyses examining the rates of GLP1-RA adherence across sociodemographic strata, including sex, race, ethnicity, income and educational attainment levels, and insurance status, are presented in eTable 3 in Supplement 1.

**Table 2.** Comparison of GLP1-RA and SGLT2i Adherence Rates by Co-payment Level

	Co-payment^a^			*P* value^b^
	Low	Medium	High	
Patients with PDC >80%, No./total No. (%)				
GLP1-RA	8407/11 689 (71.9)	7876/11 988 (65.7)	9274/15 472 (59.8)	<.001
SGLT2i	10 347/13 425 (77.1)	17 159/24 000 (71.5)	9833/13 467 (73.0)	<.001
Combination	1018/1294 (78.7)	1457/1948 (74.8)	788/1147 (68.7)	<.001
PDC, median (IQR)				
GLP1-RA	0.92 (0.77-0.99)	0.90 (0.70-0.98)	0.86 (0.67-0.96)	<.001
SGLT2i	0.95 (0.82-1.00)	0.92 (0.75-0.99)	0.92 (0.76-0.99)	<.001
Combination	0.93 (0.83-0.98)	0.91 (0.80-0.97)	0.88 (0.77-0.95)	<.001

Abbreviations: GLP1-RA, glucagon-like peptide-1 receptor agonists; PDC, proportion of days covered; SGLT2i, sodium-glucose cotransporter 2 inhibitors.

^a^ Low co-payment was defined as less than $10; medium, $10 to less than $50; and high, $50 or greater.

^b^ *P* values are based on Mantel-Haenszel χ^2^ test (categorical outcomes) or Kruskal-Wallis test (continuous outcomes).

### Adherence to SGLT2i Therapies by Co-payment Level

The final cohort comprised 51 072 individuals who had a pharmacy claim for an SGLT2i medication, including 13 425 (26.3%) with a low co-payment, 24 000 (47.0%) with a medium co-payment, and 13 647 (26.7%) with a high co-payment. Among SGLT2i users, 37 339 (73.1%) individuals achieved 12-month adherence (PDC≥80%) for SGLT2i use. Adherence varied by co-payment level, including 10 347 (77.1%) with a low-co-payment, 17 159 (71.5%) with a medium co-payment, and 9833 (72.1%) with a high co-payment having PDC of 80% or greater (*P* < .001) (Table 2). The OR changes between modeling steps by co-payment level are shown in Figure 2B. In the fully multivariable-adjusted model including demographic, clinical, and socioeconomic factors and year of index prescription fill (model 3), individuals with a medium co-payment were significantly less likely to have 12-month adherence to SGLT2i therapies compared with those with a low co-payment (AOR, 0.67; 95% CI, 0.63-0.72). Individuals with a high co-payment were also significantly less likely to have 12-month adherence to SGLT2i therapies compared with those with a low co-payment (AOR, 0.68; 95% CI, 0.63-0.72). Sensitivity analyses describing the rates of SGLT2i adherence across sociodemographic covariates, including sex, race, ethnicity, income and educational attainment levels, and insurance status, are reported in eTable 3 in Supplement 1. The adherence rates and AORs for the 4389 individuals who received both a GLP-1RA and a SGLT2i medication (ie, combination therapy) during the study period are summarized in Table 2 and eTable 3 in Supplement 1.

## Discussion

In this retrospective cohort study of 94 610 individuals with T2D and/or HF from 2014 to 2020, we observed that individuals with a lower medication co-payment had significantly higher odds of 12-month adherence to GLP1-RA and SGLT2i therapies compared with those with a higher co-payment. These differences persisted even when controlling for patient demographic, clinical, and socioeconomic covariates, demonstrating an independent association of co-payment amount with adherence to these therapies.

Using contemporary insurance claims data, our findings extend prior research that has examined various sociodemographic factors associated with the initiation of GLP1-RA and SGLT2i therapies, including race, ethnicity, household income level, and insurance status.^21,25,26^ In prior analyses using Clinformatics data through 2019, researchers found that individuals with higher median household incomes were more likely to receive an initial prescription for both a GLP1-RA and an SGLT2i.^10,11^ A similar observation was noted in the Look AHEAD trial, which determined that self-reported lower yearly family income was inversely associated with initiation of GLP1-RA and SGLT2i therapy.^27^ Others have examined alternative factors associated with GLP1-RA and SGLT2i use, such as the role of Medicare medication formulary restrictions, observing that greater access to these medications on less expensive formulary tiers was associated with greater use of these medications.^28^ A single health care system analysis of patients with T2D also found that GLP1-RA and SGLT2i nonadherence was associated with factors such as age, Black race, and higher rates of medical comorbidities.^16^ Our analysis extends upon this literature by focusing on the association of co-payment with 1-year adherence to guideline-recommended medications for chronic diseases such as T2D and HF. Our results were further strengthened by our capacity to broadly adjust for fundamental upstream social and economic variables that are associated with long-term medication adherence.

The association of co-payment with treatment adherence has been explored in both cardiovascular and noncardiovascular conditions. Examining oral anticoagulant use for atrial fibrillation management, researchers demonstrated that patients with higher medication co-payments were less likely to adhere to and more likely to discontinue these newer therapies for stroke prevention.^17^ Similar cost-sharing outcomes for adherence have been observed in oncologic conditions, which are often managed with high-cost therapies.^29,30^ In an analysis of patients with chronic myeloid leukemia, researchers found that higher co-payments were associated with lower 6-month adherence and higher discontinuation of chemotherapy.^31^ The association of out-of-pocket costs with poor medication adherence across myriad conditions suggests that patients experiencing social disadvantage, including low income or employment levels, likely struggle to access the latest guideline-recommended therapies.^32^ Our findings of low adherence among individuals with higher co-payments suggest, along with other studies,^16,17^ that out-of-pocket costs likely remain prohibitive for many patients, which will continue to contribute to inequitable access to effective therapies if not urgently addressed.

Our findings are particularly noteworthy because they were observed in a cohort of fully commercially insured individuals, suggesting that insurance access alone does not guarantee effective use of medical treatment for all patients.^22,33^ Similarly, the low-cost, uniform health system of the Veterans Health Administration has observed lower rates of GLP1-RA and SGLT2i use among certain subgroups, including individuals from underrepresented racial and ethnic groups.^21,25^ To achieve pharmacoequity, or equitable access to medications, future work is needed to examine longer-term (eg, beyond 12 months) adherence, assess how differential adherence is associated with downstream T2D and HF outcomes, and identify interventions to improve medication adherence for individuals with high co-payments. Such interventions may need to extend beyond addressing well-described patient and practitioner behaviors to target policy-based solutions that reduce high medication costs, including for GLP1-RA and SGLT2i therapies.^33,34^ Elimination or substantial lowering of out-of-pocket costs has been demonstrated in adjacent disciplines of cardiovascular diseases to be associated with improved medication adherence, while not influencing overall health system costs.^35^

### Strengths and Limitations

Our study findings were strengthened by the ability to assess medication use in a large, nationally representative sample of insured individuals with increasingly morbid cardiometabolic conditions. However, there are limitations to our study. First, we were unable to exclude residual confounding from any unmeasured individual-level social factors, which may confound the association between co-payment and medication adherence. Second, there may be concerns about the generalizability of an analysis of commercially insured individuals to those without health insurance or with public insurance. However, as demonstrated, even among those with health insurance, the variability of co-payment was associated with reduced medication adherence, which may be even more pronounced among those who are underinsured or uninsured. Third, we did not capture individual patient preferences associated with medication use, including specific reasons for poor medication adherence. We also did not assess other baseline medications received or the amount spent on these medications, switching from 1 GLP1-RA or SGLT2i therapy to another, or medication discontinuation or nonpersistence rates. We also did not examine practitioner decision-making associated with medication prescribing. Fourth, as claims data exist primarily for billing and reimbursement, we could not exclude misclassification of T2D or HF diagnoses or medical comorbidities; however, we expect such misclassification to be nondifferential and therefore bias our assessments toward the null. Fifth, co-payment information was only available for those who filled a prescription; thus, we were unable to assess how co-payment influenced initial prescription receipt or abandonment at the pharmacy. Complementing this avenue of limitations, we were also unable to assess other unique factors associated with adherence, such as price inflation.

## Conclusions

In this cohort study of individuals with T2D and/or HF, we found that medication co-payment was independently associated with 12-month adherence to GLP1-RA and SGLT2i medications even after adjustment for sociodemographic and clinical factors. This finding in a commercially insured cohort has important implications for ensuring equitable access to medical management of chronic cardiometabolic diseases. Lowering high out-of-pocket prescription costs may be key to improving adherence to guideline-recommended therapies and advancing overall quality of care in T2D and HF management.

## Notes

Supplement 1. **eTable 1.** *ICD-9-CM*, *ICD-10-CM*, and *CPT* Codes Used to Determine Comorbidities **eTable 2.** Characteristics of Individuals Using Combination SGLT2i and GLP1-RA Therapy by Co-payment Level **eTable 3.** Adherence Rates to GLP1-RA or SGLT2i Therapy, Adjusted for Sociodemographic Factors and Stratified by Co-payment Status

Supplement 2.
Data Sharing Statement
