# Supplementary material for: Association of Prescription Co-payment With Adherence to Glucagon-Like Peptide-1 Receptor Agonist and Sodium-Glucose Cotransporter-2 Inhibitor Therapies in Patients With Heart Failure and Diabetes
Source: JAMA Netw Open. 2023 Jun 1;6(6):e2316290. doi: 10.1001/jamanetworkopen.2023.16290 (PMC10236237; doi:10.1001/jamanetworkopen.2023.16290)
Supplement: Supplement 1. — eTable 1. ICD-9-CM, ICD-10-CM, and CPT Codes Used to Determine Comorbidities eTable 2. Characteristics of Individuals Using Combination SGLT2i and GLP1-RA Therapy by Co-payment Level eTable 3. Adherence Rates to GLP1-RA or SGLT2i Therapy, Adjusted for Sociodemographic Factors and Stratified by Co-payment Status [file jamanetwopen-e2316290-s001.pdf]

Supplementary Online Content

Essien UR, Singh B, Swabe G, et al. Association of prescription co-payment with adherence to glucagon-like peptide-1 receptor agonist and sodium-glucose cotransporter-2 inhibitor therapies in patients with heart failure and diabetes. *JAMA Netw Open*. 2023;6(6):e2316290. doi:10.1001/jamanetworkopen.2023.16290

eTable 1. *ICD-9-CM*, *ICD-10-CM*, and *CPT* Codes Used to Determine Comorbidities
eTable 2. Characteristics of Individuals Using Combination SGLT2i and GLP1-RA Therapy by Co-payment Level
eTable 3. Adherence Rates to GLP1-RA or SGLT2i Therapy, Adjusted for Sociodemographic Factors and Stratified by Co-payment Status

This supplementary material has been provided by the authors to give readers additional information about their work.

© 2023 Essien UR et al. *JAMA Network Open*.

**eTable 1:** ICD-9-CM, ICD-10-CM, and CPT codes used to determine comorbidities	
**Condition**	**ICD-9-CM, ICD-10-CM, and CPT Codes**
Congestive Heart Failure	I09.81,I11.0,I13.0,I13.2,I50.1,I50.20,I50.21,I50.22,I50.23,I50.30,I50.31,I50.32,I50.33,I50.40,I50.41,I50.42,I50.43,I50.810,I50.811,I50.812,I50.813,I50.814,I50.82,I50.83,I50.84,I50.89,I50.9,I51.81,I97.130,I97.131,O29.121,O29.122,O29.123,O29.129,R57.0,Z95.811,Z95.812,398.91,402.01,402.11,402.91,404.01,404.03,404.11,404.13,404.91,404.93,428.0,428.1,428.2,428.3,428.4,428.5,428.6,428.7,428.8,428.9
Valvular disease	A18.84,A32.82,A39.51,A52.03,B33.21,B37.6,I01.1,I01.8,I01.9,I02.0,I05.0,I05.1,I05.2,I05.8,I05.9,I06.0,I06.1,I06.2,I06.8,I06.9,I07.0,I07.1,I07.2,I07.8,I07.9,I08.0,I08.1,I08.2,I08.3,I08.8,I08.9,I09.1,I09.89,I33.0,I33.9,I34.0,I34.1,I34.2,I34.8,I34.9,I35.0,I35.1,I35.2,I35.8,I35.9,I36.0,I36.1,I36.2,I36.8,I36.9,I37.0,I37.1,I37.2,I37.8,I37.9,I38.X,I39.X,M32.11,Q22.0,Q22.1,Q22.2,Q22.3,Q22.4,Q22.5,Q22.6,Q22.8,Q22.9,Q23.0,Q23.1,Q23.2,Q23.3,Q23.4,Q23.8,Q23.9,T82.01XA,T82.01XD,T82.01XS,T82.02XA,T82.02XD,T82.02XS,T82.03XA,T82.03XD,T82.03XS,T82.09XA,T82.09XD,T82.09XS,T82.221A,T82.221D,T82.221S,T82.222A,T82.222D,T82.222S,T82.223A,T82.223D,T82.223S,T82.228A,T82.228D,T82.228S,T82.6XXA,T82.6XXD,T82.6XXS,Z95.2,Z95.3,Z95.4,932.0,932.1,932.2,932.3,932.4,394.0,394.1,394.2,394.3,394.4,394.5,394.6,394.7,394.8,394.9,395.0,395.1,395.2,395.3,395.4,395.5,395.6,395.7,395.8,395.9,396.0,396.1,396.2,396.3,396.4,396.5,396.6,396.7,396.8,396.9,397.0,397.1,397.9,424.0,424.01,424.02,424.03,424.04,424.05,424.06,424.07,424.08,424.09,424.10,424.11,424.12,424.13,424.14,424.15,424.16,424.17,424.18,424.19,424.20,424.21,424.22,424.23,424.24,424.25,424.26,424.27,424.28,424.29,424.30,424.31,424.32,424.33,424.34,424.35,424.36,424.37,424.38,424.39,424.40,424.41,424.42,424.43,424.44,424.45,424.46,424.47,424.48,424.49,424.50,424.51,424.52,424.53,424.54,424.55,424.56,424.57,424.58,424.59,424.60,424.61,424.62,424.63,424.64,424.65,424.66,424.67,424.68,424.69,424.70,424.71,424.72,424.73,424.74,424.75,424.76,424.77,424.78,424.79,424.80,424.81,424.82,424.83,424.84,424.85,424.86,424.87,424.88,424.89,424.90,424.91,424.92,424.93,424.94,424.95,424.96,424.97,424.98,424.99,746.3,746.4,746.5,746.6,V42.2,V43.3
Pulmonary circulation disorders	I27.0,I27.1,I27.2,I27.20,I27.21,I27.22,I27.23,I27.24,I27.29,I27.81,I27.82,I27.83,I27.89,I27.9,I28.0,I28.1,I28.8,I28.9,415.11,415.12,415.13,415.14,415.15,415.16,415.17,415.18,415.19,416.0,416.1,416.2,416.3,416.4,416.5,416.6,416.7,416.8,416.9,417.9
Peripheral vascular disorders	A52.00,A52.01,A52.02,A52.09,I70.0,I70.1,I70.201,I70.202,I70.203,I70.208,I70.209,I70.211,I70.212,I70.213,I70.218,I70.219,I70.221,I70.222,I70.223,I70.228,I70.229,I70.231,I70.232,I70.233,I70.234,I70.235,I70.238,I70.239,I70.241,I70.242,I70.243,I70.244,I70.245,I70.248,I70.249,I70.25,I70.261,I70.262,I70.263,I70.268,I70.269,I70.291,I70.292,I70.293,I70.298,I70.299,I70.301,I70.302,I70.303,I70.308,I70.309,I70.311,I70.312,I70.313,I70.318,I70.319,I70.321,I70.322,I70.323,I70.328,I70.329,I70.331,I70.332,I70.333,I70.334,I70.335,I70.338,I70.339,I70.341,I70.342,I70.343,I70.344,I70.345,I70.348,I70.349,I70.35,I70.361,I70.362,I70.363,I70.368,I70.369,I70.391,I70.392,I70.393,I70.398,I70.399,I70.401,I70.402,I70.403,I70.408,I70.409,I70.411,I70.412,I70.413,I70.418,I70.419,I70.421,I70.422,I70.423,I70.428,I70.429,I70.431,I70.432,I70.433,I70.434,I70.435,I70.438,I70.439,I70.441,I70.442,I70.443,I70.444,I70.445,I70.448,I70.449,I70.45,I70.461,I70.462,I70.463,I70.468,I70.469,I70.491,I70.492,I70.493,I70.498,I70.499,I70.501,I70.502,I70.503,I70.508,I70.509,I70.511,I70.512,I70.513,I70.518,I70.519,I70.521,I70.522,I70.523,I70.528,I70.529,I70.531,I70.532,I70.533,I70.534,I70.535,I70.538,I70.539,I70.541,I70.542,I70.543,I70.544,I70.545,I70.548,I70.549,I70.55,I70.561,I70.562,I70.563,I70.568,I70.569,I70.591,I70.592,I70.593,I70.598,I70.599,I70.601,I70.602,I70.603,I70.608,I70.609,I70.611,I70.612,I70.613,I70.618,I70.619,I70.621,I70.622,I70.623,I70.628,I70.629,I70.631,I70.632,I70.633,I70.634,I70.635,I70.638,I70.639,I70.641,I70.642,I70.643,I70.644,I70.645,I70.648,I70.649,I70.65,I70.661,I70.662,I70.663,I70.668,I70.669,I70.691,I70.692,I70.693,I70.698,I70.699,I70.701,I70.702,I70.703,I70.708,I70.709,I70.711,I70.712,I70.713,I70.718,I70.719,I70.721,I70.722,I70.723,I70.728,I70.729,I70.731,I70.732,I70.733,I70.734,I70.735,I70.738,I70.739,I70.741,I70.742,I70.743,I70.744,I70.745,I70.748,I70.749,I70.75,I70.761,I70.762,I70.763,I70.768,I70.769,I70.791,I70.792,I70.793,I70.798,I70.799,I70.8,I70.90,I70.91,I70.92,I71.00,I71.01,I71.02,I71.03,I71.1,I71.2,I71.3,I71.4,I71.5,I71.6,I71.8,I71.9,I72.0,I72.1,I72.2,I72.3,I72.4,I72.5,I72.6,I72.8,I72.9,I73.01,I73.1,I73.81,I73.89,I73.9,I74.01,I74.09,I74.10,I74.11,I74.19,I74.2,I74.3,I74.4,I74.5,I74.8,I74.9,I75.011,I75.012,I75.013,I75.019,I75.021,I75.022,I75.023,I75.029,I75.81,I75.89,I77.0,I77.1,I77.2,I77.3,I77.4,I77.5,I77.6,I77.70,I77.71,I77.72,I77.73,I77.74,I77.75,I77.76,I77.77,I77.79,I77.810,I77.811,I77.812,I77.819,I77.89,I77.9,I78.0,I78.1,I78.8,I78.9,I79.0,I79.1,I79.8,I99.8,I99.9,K31.811,K31.819,K55.1,K55.8,K55.9,Z95.820,Z95.828,440.X,440.1,440.2,440.3,440.4,440.5,440.6,440.7,440.8,440.9,441.00,441.01,441.02,441.03,441.04,441.05,441.06,441.07,441.08,441.09,441.10,441.11,441.12,441.13,441.14,441.15,441.16,441.17,441.18,441.19,441.20,441.21,441.22,441.23,441.24,441.25,441.26,441.27,441.28,441.29,441.30,441.31,441.32,441.33,441.34,441.35,441.36,441.37,441.38,441.39,441.40,441.41,441.42,441.43,441.44,441.45,441.46,441.47,441.48,441.49,441.50,441.51,441.52,

© 2023 Essien UR et al. *JAMA Network Open*.

Condition	ICD-9-CM, ICD-10-CM, and CPT Codes
Peripheral vascular disorders (*continued*)	441.53,441.54,441.55,441.56,441.57,441.58,441.59,441.60,441.61,441.62,441.63,441.64,441.65,441.66,441.67,441.68,441.69,441.70,441.71,441.72,441.73,441.74,441.75,441.76,441.77,441.78,441.79,441.80,441.81,441.82,441.83,441.84,441.85,441.86,441.87,441.88,441.89,441.90,442.0,442.1,442.2,442.3,442.4,442.5,442.6,442.7,442.8,442.9,443.1,443.2,443.3,443.4,443.5,443.6,443.7,443.8,443.9,444.21,444.22,444.71,449.X,557.1,557.9,V43.4
Hypertension (uncomplicated)	I10.X,I16.0,I16.9,O10.011,O10.012,O10.013,O10.019,O10.02,O10.03,401.1,401.9,642.00,642.01,642.02,642.03,642.04
Hypertension (complicated)	H35.031,H35.032,H35.033,H35.039,I11.0,I11.9,I12.0,I12.9,I13.0,I13.10,I13.11,I13.2,I15.0,I15.1,I15.2,I15.8,I15.9,I16.1,I67.4,O10.111,O10.112,O10.113,O10.119,O10.12,O10.13,O10.211,O10.212,O10.213,O10.219,O10.22,O10.23,O10.311,O10.312,O10.313,O10.319,O10.32,O10.33,O10.411,O10.412,O10.413,O10.419,O10.42,O10.43,O10.911,O10.912,O10.913,O10.919,O10.92,O10.93,O11.1,O11.2,O11.3,O11.4,O11.5,O11.9,O16.1,O16.2,O16.3,O16.4,O16.5,O16.9,401.0,402.00,402.01,402.02,402.03,402.04,402.05,402.06,402.07,402.08,402.09,402.10,402.11,402.12,402.13,402.14,402.15,402.16,402.17,402.18,402.19,402.20,402.21,402.22,402.23,402.24,402.25,402.26,402.27,402.28,402.29,402.30,402.31,402.32,402.33,402.34,402.35,402.36,402.37,402.38,402.39,402.40,402.41,402.42,402.43,402.44,402.45,402.46,402.47,402.48,402.49,402.50,402.51,402.52,402.53,402.54,402.55,402.56,402.57,402.58,402.59,402.60,402.61,402.62,402.63,402.64,402.65,402.66,402.67,402.68,402.69,402.70,402.71,402.72,402.73,402.74,402.75,402.76,402.77,402.78,402.81,402.82,402.83,402.84,402.85,402.86,402.87,402.88,402.89,402.90,402.91,402.92,402.93,402.94,402.95,402.96,402.97,402.98,402.99,403.00,403.01,403.02,403.03,403.04,403.05,403.06,403.07,403.08,403.09,403.10,403.11,403.12,403.13,403.14,403.15,403.16,403.17,403.18,403.19,403.20,403.21,403.22,403.23,403.24,403.25,403.26,403.27,403.28,403.29,403.30,403.31,403.32,403.33,403.34,403.35,403.36,403.37,403.38,403.39,403.40,403.41,403.42,403.43,403.44,403.45,403.46,403.47,403.48,403.49,403.50,403.51,403.52,403.53,403.54,403.55,403.56,403.57,403.58,403.59,403.60,403.61,403.62,403.63,403.64,403.65,403.66,403.67,403.68,403.69,403.70,403.71,403.72,403.73,403.74,403.75,403.76,403.77,403.78,403.79,403.80,403.81,403.82,403.83,403.84,403.85,403.86,403.87,403.88,403.89,403.90,403.91,403.92,403.93,403.94,403.95,403.96,403.97,403.98,403.99,404.00,404.01,404.02,404.03,404.04,404.05,404.06,404.07,404.08,404.09,404.10,404.11,404.12,404.13,404.14,404.15,404.16,404.17,404.18,404.19,404.20,404.21,404.22,404.23,404.24,404.25,404.26,404.27,404.28,404.29,404.30,404.31,404.32,404.33,404.34,404.35,404.36,404.37,404.38,404.39,404.40,404.41,404.42,404.43,404.44,404.45,404.46,404.47,404.48,404.49,404.50,404.51,404.52,404.53,404.54,404.55,404.56,404.57,404.58,404.59,404.60,404.61,404.62,404.63,404.64,404.65,404.66,404.67,404.68,404.69,404.70,404.71,404.72,404.73,404.74,404.75,404.76,404.77,404.78,404.79,404.80,404.81,404.82,404.83,404.84,404.85,404.86,404.87,404.88,404.89,404.90,404.91,404.92,404.93,404.94,404.95,404.96,404.97,404.98,404.99,405.00,405.01,405.02,405.03,405.04,405.05,405.06,405.07,405.08,405.09,405.10,405.11,405.12,405.13,405.14,405.15,405.16,405.17,405.18,405.19,405.20,405.21,405.22,405.23,405.24,405.25,405.26,405.27,405.28,405.29,405.30,405.31,405.32,405.33,405.34,405.35,405.36,405.37,405.38,405.39,405.40,405.41,405.42,405.43,405.44,405.45,405.46,405.47,405.48,405.49,405.50,405.51,405.52,405.53,405.54,405.55,405.56,405.57,405.58,405.59,405.60,405.61,405.62,405.63,405.64,405.65,405.66,405.67,405.68,405.69,405.70,405.71,405.72,405.73,405.74,405.75,405.76,405.77,405.78,405.79,405.80,405.81,405.82,405.83,405.84,405.85,405.86,405.87,405.88,405.89,405.90,405.91,405.92,405.93,405.94,405.95,405.96,405.97,405.98,405.99,437.2,642.10,642.11,642.12,642.13,642.14,642.15,642.16,642.17,642.18,642.19,642.20,642.21,642.22,642.23,642.24,642.70,642.71,642.72,642.73,642.74,642.75,642.76,642.77,642.78,642.79,642.80,642.81,642.82,642.83,642.84,642.85,642.86,642.87,642.88,642.89,642.90,642.91,642.92,642.93,642.94
Paralysis	G04.1,G80.0,G80.1,G80.2,G80.8,G80.9,G81.00,G81.01,G81.02,G81.03,G81.04,G81.10,G81.11,G81.12,G81.13,G81.14,G81.90,G81.91,G81.92,G81.93,G81.94,G82.20,G82.21,G82.22,G82.50,G82.51,G82.52,G82.53,G82.54,G83.0,G83.10,G83.11,G83.12,G83.13,G83.14,G83.20,G83.21,G83.22,G83.23,G83.24,G83.30,G83.31,G83.32,G83.33,G83.34,G83.4,G83.5,G83.81,G83.82,G83.83,G83.84,G83.89,G83.9,I69.031,I69.032,I69.033,I69.034,I69.039,I69.041,I69.042,I69.043,I69.044,I69.049,I69.051,I69.052,I69.053,I69.054,I69.059,I69.061,I69.062,I69.063,I69.064,I69.065,I69.069,I69.131,I69.132,I69.133,I69.134,I69.139,I69.141,I69.142,I69.143,I69.144,I69.149,I69.151,I69.152,I69.153,I69.154,I69.159,I69.161,I69.162,I69.163,I69.164,I69.165,I69.169,I69.231,I69.232,I69.233,I69.234,I69.239,I69.241,I69.242,I69.243,I69.244,I69.249,I69.251,I69.252,I69.253,I69.254,I69.259,I69.261,I69.262,I69.263,I69.264,I69.265,I69.269,I69.331,I69.332,I69.333,I69.334,I69.339,I69.341,I69.342,I69.343,I69.344,I69.349,I69.351,I69.352,I69.353,I69.354,I69.359,I69.361,I69.362,I69.363,I69.364,I69.365,I69.369,I69.831,I69.832,I69.833,I69.834,I69.839,I69.841,I69.842,I69.843,I69.844,I69.849,I69.851,I69.852,I69.853,I69.854,I69.859,I69.861,I69.862,I69.863,I69.864,I69.865,I69.869,I69.931,I69.932,I69.933,I69.934,I69.939,I69.941,I69.942,I69.

© 2023 Essien UR et al. *JAMA Network Open*.

Condition	ICD-9-CM, ICD-10-CM, and CPT Codes
Paralysis (*continued*)	943,I69.944,I69.949,I69.951,I69.952,I69.953,I69.954,I69.959,I69.961,I69.962,I69.963,I69.964,I69.965,I69.969,R53.2,342.0,342.1,342.2,342.3,342.4,342.5,342.6,342.7,342.8,342.9,343.0,343.1,343.2,343.3,343.4,343.5,343.6,343.7,343.8,343.9,344.0,344.1,344.2,344.3,344.4,344.5,344.6,344.7,344.8,344.9,438.20,438.21,438.22,438.23,438.24,438.25,438.26,438.27,438.28,438.29,438.30,438.31,438.32,438.33,438.34,438.35,438.36,438.37,438.38,438.39,438.40,438.41,438.42,438.43,438.44,438.45,438.46,438.47,438.48,438.49,438.50,438.51,438.52,438.53,780.72
Neurological disorders affecting movement	G08.X,G10.X,G11.0,G11.1,G11.10,G11.11,G11.19,G11.2,G11.3,G11.4,G11.8,G11.9,G12.0,G12.1,G12.20,G12.21,G12.22,G12.23,G12.24,G12.25,G12.29,G12.8,G12.9,G13.0,G13.1,G13.2,G13.8,G20.X,G21.0,G21.11,G21.19,G21.2,G21.3,G21.4,G21.8,G21.9,G23.0,G23.1,G23.2,G23.8,G23.9,G24.09,G24.1,G24.2,G24.8,G25.4,G25.5,G25.70,G25.71,G25.79,G25.81,G25.82,G25.83,G25.89,G25.9,G26.X,G32.0,G32.81,G32.89,G80.3
Seizures and epilepsy	G40.001,G40.009,G40.011,G40.019,G40.101,G40.109,G40.111,G40.119,G40.201,G40.209,G40.211,G40.219,G40.301,G40.309,G40.311,G40.319,G40.401,G40.409,G40.411,G40.419,G40.42,G40.501,G40.509,G40.801,G40.802,G40.803,G40.804,G40.811,G40.812,G40.813,G40.814,G40.821,G40.822,G40.823,G40.824,G40.833,G40.834,G40.89,G40.901,G40.909,G40.911,G40.919,G40.A01,G40.A09,G40.A11,G40.A19,G40.B01,G40.B09,G40.B11,G40.B19,R56.1,R56.9
Other neurological disorders	E75.00,E75.01,E75.02,E75.09,E75.10,E75.11,E75.19,E75.23,E75.25,E75.26,E75.29,E75.4,F05.X,F84.2,G35.X,G36.0,G36.8,G36.9,G37.0,G37.1,G37.2,G37.3,G37.4,G37.5,G37.8,G37.9,G47.411,G47.419,G47.421,G47.429,G89.0,G91.0,G91.1,G91.2,G91.3,G91.4,G91.8,G91.9,G93.0,G93.40,G93.41,G93.49,G93.5,G93.6,G93.7,G93.81,G93.82,G93.89,G93.9,G94.X,O99.350,O99.351,O99.352,O99.353,O99.354,O99.355,P91.60,P91.61,P91.62,P91.63,330.1,330.2,330.3,330.4,330.5,330.6,330.7,330.8,330.9,331.0,331.1,331.2,331.3,331.4,331.5,331.6,331.7,331.8,331.9,332.0,333.4,333.5,333.71,333.72,333.79,333.85,333.94,334.0,334.1,334.2,334.3,334.4,334.5,334.6,334.7,334.8,334.9,335.0,335.1,335.2,335.3,335.4,335.5,335.6,335.7,335.8,335.9,338.0,340.X,341.1,341.2,341.3,341.4,341.5,341.6,341.7,341.8,341.9,345.00,345.01,345.02,345.03,345.04,345.05,345.06,345.07,345.08,345.09,345.10,345.11,345.2,345.3,345.40,345.41,345.42,345.43,345.44,345.45,345.46,345.47,345.48,345.49,345.50,345.51,345.52,345.53,345.54,345.55,345.56,345.57,345.58,345.59,345.60,345.61,345.62,345.63,345.64,345.65,345.66,345.67,345.68,345.69,345.70,345.71,345.72,345.73,345.74,345.75,345.76,345.77,345.78,345.79,345.80,345.81,345.82,345.83,345.84,345.85,345.86,345.87,345.88,345.89,345.90,345.91,347.00,347.01,347.10,347.11,649.40,649.41,649.42,649.43,649.44,768.7,768.70,768.71,768.72,780.3,780.31,780.32,780.33,780.39,780.97,784.3
Chronic pulmonary disease	J41.0,J41.1,J41.8,J42.X,J43.0,J43.1,J43.2,J43.8,J43.9,J44.0,J44.1,J44.9,J45.20,J45.21,J45.22,J45.30,J45.31,J45.32,J45.40,J45.41,J45.42,J45.50,J45.51,J45.52,J45.901,J45.902,J45.909,J45.990,J45.991,J45.998,J47.0,J47.1,J47.9,J60.X,J61.X,J62.0,J62.8,J63.0,J63.1,J63.2,J63.3,J63.4,J63.5,J63.6,J64.X,J65.X,J66.0,J66.1,J66.2,J66.8,J67.0,J67.1,J67.2,J67.3,J67.4,J67.5,J67.6,J67.7,J67.8,J67.9,J68.4,J70.1,J70.3,490.X,490.1,490.2,490.3,490.4,490.5,490.6,490.7,490.8,490.9,491.0,491.1,491.2,491.3,491.4,491.5,491.6,491.7,491.8,491.9,492.0,492.1,492.2,492.3,492.4,492.5,492.6,492.7,492.8,493.00,493.01,493.02,493.03,493.04,493.05,493.06,493.07,493.08,493.09,493.10,493.11,493.12,493.13,493.14,493.15,493.16,493.17,493.18,493.19,493.20,493.21,493.22,493.23,493.24,493.25,493.26,493.27,493.28,493.29,493.30,493.31,493.32,493.33,493.34,493.35,493.36,493.37,493.38,493.39,493.40,493.41,493.42,493.43,493.44,493.45,493.46,493.47,493.48,493.49,493.50,493.51,493.52,493.53,493.54,493.55,493.56,493.57,493.58,493.59,493.60,493.61,493.62,493.63,493.64,493.65,493.66,493.67,493.68,493.69,493.70,493.71,493.72,493.73,493.74,493.75,493.76,493.77,493.78,493.79,493.80,493.81,493.82,493.83,493.84,493.85,493.86,493.87,493.88,493.89,493.90,493.91,493.92,494.X,494.0,494.1,495.0,495.1,495.2,495.3,495.4,495.5,495.6,495.7,495.8,495.9,496.0,496.1,496.2,496.3,496.4,496.5,496.6,496.7,496.8,496.9,497.0,497.1,497.2,497.3,497.4,497.5,497.6,497.7,497.8,497.9,498.0,498.1,498.2,498.3,498.4,498.5,498.6,498.7,498.8,498.9,499.0,499.1,499.2,499.3,499.4,499.5,499.6,499.7,499.8,499.9,500.0,500.1,500.2,500.3,500.4,500.5,500.6,500.7,500.8,500.9,501.0,501.1,501.2,501.3,501.4,501.5,501.6,501.7,501.8,501.9,502.0,502.1,502.2,502.3,502.4,502.5,502.6,502.7,502.8,502.9,503.0,503.1,503.2,503.3,503.4,503.5,503.6,503.7,503.8,503.9,504.0,504.1,504.2,504.3,504.4,504.5,504.6,504.7,504.8,504.9,505.X,506.4
Diabetes (uncomplicated)	E08.21,E08.22,E08.29,E08.311,E08.319,E08.321,E08.3211,E08.3212,E08.3213,E08.3219,E08.329,E08.3291,E08.3292,E08.3293,E08.3299,E08.331,E08.3311,E08.3312,E08.3313,E08.3319,E08.339,E08.3391,E08.3392,E08.3393,E08.3399,E08.341,E08.3411,E08.3412,E08.3413,E08.3419,E08.349,E08.3491,E08.3492,E08.3493,E08.3499,E08.351,E08.3511,E08.3512,E08.3513,E08.3519,E08.3521,E08.3522,E08.3523,E08.3529,E08.3531,E08.3532,E08.3533,E08.3539,E08.3541,E08.3542,E08.3543,E08.3549,E08.3551,E08.3552,E08.3553,E08.3559,E08.359,E08.3591,E08.3592,E08.3593,E08.3599,E08.36,E08.37X1,E08.37X2,E08.37X3,E08.37X9,

Condition	ICD-9-CM, ICD-10-CM, and CPT Codes
Diabetes (uncomplicated, *continued*)	E08.39,E08.40,E08.41,E08.42,E08.43,E08.44,E08.49,E08.51,E08.52,E08.59,E08.610,E08.618,E08.620,E08.621,E08.622,E08.628,E08.630,E08.638,E08.641,E08.649,E08.65,E08.69,E08.8,E09.21,E09.22,E09.29,E09.311,E09.319,E09.321,E09.3211,E09.3212,E09.3213,E09.3219,E09.329,E09.3291,E09.3292,E09.3293,E09.3299,E09.331,E09.3311,E09.3312,E09.3313,E09.3319,E09.339,E09.3391,E09.3392,E09.3393,E09.3399,E09.341,E09.3411,E09.3412,E09.3413,E09.3419,E09.349,E09.3491,E09.3492,E09.3493,E09.3499,E09.351,E09.3511,E09.3512,E09.3513,E09.3519,E09.3521,E09.3522,E09.3523,E09.3529,E09.3531,E09.3532,E09.3533,E09.3539,E09.3541,E09.3542,E09.3543,E09.3549,E09.3551,E09.3552,E09.3553,E09.3559,E09.359,E09.3591,E09.3592,E09.3593,E09.3599,E09.36,E09.37X1,E09.37X2,E09.37X3,E09.37X9,E09.39,E09.40,E09.41,E09.42,E09.43,E09.44,E09.49,E09.51,E09.52,E09.59,E09.610,E09.618,E09.620,E09.621,E09.622,E09.628,E09.630,E09.638,E09.641,E09.649,E09.65,E09.69,E09.8,E10.21,E10.22,E10.29,E10.311,E10.319,E10.321,E10.3211,E10.3212,E10.3213,E10.3219,E10.329,E10.3291,E10.3292,E10.3293,E10.3299,E10.331,E10.3311,E10.3312,E10.3313,E10.3319,E10.339,E10.3391,E10.3392,E10.3393,E10.3399,E10.341,E10.3411,E10.3412,E10.3413,E10.3419,E10.349,E10.3491,E10.3492,E10.3493,E10.3499,E10.351,E10.3511,E10.3512,E10.3513,E10.3519,E10.3521,E10.3522,E10.3523,E10.3529,E10.3531,E10.3532,E10.3533,E10.3539,E10.3541,E10.3542,E10.3543,E10.3549,E10.3551,E10.3552,E10.3553,E10.3559,E10.359,E10.3591,E10.3592,E10.3593,E10.3599,E10.36,E10.37X1,E10.37X2,E10.37X3,E10.37X9,E10.39,E10.40,E10.41,E10.42,E10.43,E10.44,E10.49,E10.51,E10.52,E10.59,E10.610,E10.618,E10.620,E10.621,E10.622,E10.628,E10.630,E10.638,E10.641,E10.649,E10.65,E10.69,E10.8,E11.21,E11.22,E11.29,E11.311,E11.319,E11.321,E11.3211,E11.3212,E11.3213,E11.3219,E11.329,E11.3291,E11.3292,E11.3293,E11.3299,E11.331,E11.3311,E11.3312,E11.3313,E11.3319,E11.339,E11.3391,E11.3392,E11.3393,E11.3399,E11.341,E11.3411,E11.3412,E11.3413,E11.3419,E11.349,E11.3491,E11.3492,E11.3493,E11.3499,E11.351,E11.3511,E11.3512,E11.3513,E11.3519,E11.3521,E11.3522,E11.3523,E11.3529,E11.3531,E11.3532,E11.3533,E11.3539,E11.3541,E11.3542,E11.3543,E11.3549,E11.3551,E11.3552,E11.3553,E11.3559,E11.359,E11.3591,E11.3592,E11.3593,E11.3599,E11.36,E11.37X1,E11.37X2,E11.37X3,E11.37X9,E11.39,E11.40,E11.41,E11.42,E11.43,E11.44,E11.49,E11.51,E11.52,E11.59,E11.610,E11.618,E11.620,E11.621,E11.622,E11.628,E11.630,E11.638,E11.641,E11.649,E11.65,E11.69,E11.8,E13.21,E13.22,E13.29,E13.311,E13.319,E13.321,E13.3211,E13.3212,E13.3213,E13.3219,E13.329,E13.3291,E13.3292,E13.3293,E13.3299,E13.331,E13.3311,E13.3312,E13.3313,E13.3319,E13.339,E13.3391,E13.3392,E13.3393,E13.3399,E13.341,E13.3411,E13.3412,E13.3413,E13.3419,E13.349,E13.3491,E13.3492,E13.3493,E13.3499,E13.351,E13.3511,E13.3512,E13.3513,E13.3519,E13.3521,E13.3522,E13.3523,E13.3529,E13.3531,E13.3532,E13.3533,E13.3539,E13.3541,E13.3542,E13.3543,E13.3549,E13.3551,E13.3552,E13.3553,E13.3559,E13.359,E13.3591,E13.3592,E13.3593,E13.3599,E13.36,E13.37X1,E13.37X2,E13.37X3,E13.37X9,E13.39,E13.40,E13.41,E13.42,E13.43,E13.44,E13.49,E13.51,E13.52,E13.59,E13.610,E13.618,E13.620,E13.621,E13.622,E13.628,E13.630,E13.638,E13.641,E13.649,E13.65,E13.69,E13.8,249.00,249.01,249.02,249.03,249.04,249.05,249.06,249.07,249.08,249.09,249.10,249.11,249.12,249.13,249.14,249.15,249.16,249.17,249.18,249.19,249.20,249.21,249.22,249.23,249.24,249.25,249.26,249.27,249.28,249.29,249.30,249.31,250.00,250.01,250.02,250.03,250.04,250.05,250.06,250.07,250.08,250.09,250.10,250.11,250.12,250.13,250.14,250.15,250.16,250.17,250.18,250.19,250.20,250.21,250.22,250.23,250.24,250.25,250.26,250.27,250.28,250.29,250.30,250.31,250.32,250.33,648.00,648.01,648.02,648.03,648.04
Diabetes (complicated)	E08.00,E08.01,E08.10,E08.11,E08.9,E09.00,E09.01,E09.10,E09.11,E09.9,E10.10,E10.11,E10.9,E11.00,E11.01,E11.10,E11.11,E11.9,E13.00,E13.01,E13.10,E13.11,E13.9,O24.011,O24.012,O24.013,O24.019,O24.02,O24.03,O24.111,O24.112,O24.113,O24.119,O24.12,O24.13,O24.311,O24.312,O24.313,O24.319,O24.32,O24.33,O24.410,O24.414,O24.415,O24.419,O24.420,O24.424,O24.425,O24.429,O24.430,O24.434,O24.435,O24.439,O24.811,O24.812,O24.813,O24.819,O24.82,O24.83,O24.911,O24.912,O24.913,O24.919,O24.92,O24.93,249.40,249.41,249.42,249.43,249.44,249.45,249.46,249.47,249.48,249.49,249.50,249.51,249.52,249.53,249.54,249.55,249.56,249.57,249.58,249.59,249.60,249.61,249.62,249.63,249.64,249.65,249.66,249.67,249.68,249.69,249.70,249.71,249.72,249.73,249.74,249.75,249.76,249.77,249.78,249.79,249.80,249.81,249.82,249.83,249.84,249.85,249.86,249.87,249.88,249.89,249.90,249.91,250.40,250.41,250.42,250.43,250.44,250.45,250.46,250.47,250.48,250.49,250.50,250.51,250.52,250.53,250.54,250.55,250.56,250.57,250.58,250.59,250.60,250.61,250.62,250.63,250.64,250.65,250.66,250.67,250.68,250.69,250.70,250.71,250.72,250.73,250.74,250.75,250.76,250.77,250.78,250.79,250.80,250.81,250.82,250.83,250.84,250.85,250.86,250.87,250.88,250.89,250.90,250.91,250.92,250.93,775.1

© 2023 Essien UR et al. *JAMA Network Open*.

Condition	ICD-9-CM, ICD-10-CM, and CPT Codes
Hypothyroidism	E00.0,E00.1,E00.2,E00.9,E01.0,E01.1,E01.2,E01.8,E02.X,E03.0,E03.1,E03.2,E03.3,E03.4,E03.5,E03.8,E03.9,E89.0,243.0,243.1,243.2,243.3,243.4,243.5,243.6,243.7,243.8,243.9,244.0,244.1,244.2,244.8,244.9
Renal failure (moderate)	N18.3,N18.30,N18.31,N18.32,N18.9,N19.X,403.01,403.11,403.91,402.X,404.03,404.12,404.13,404.92,404.93,585.3,585.4,585.5,585.6,585.9,586.X,V42.0,V45.1,V45.11,V45.12,V56.0,V56.00,V56.01,V56.02,V56.03,V56.04,V56.05,V56.06,V56.07,V56.08,V56.09,V56.10,V56.11,V56.12,V56.13,V56.14,V56.15,V56.16,V56.17,V56.18,V56.19,V56.20,V56.21,V56.22,V56.23,V56.24,V56.25,V56.26,V56.27,V56.28,V56.29,V56.30,V56.31,V56.32,V56.8
Renal failure (severe)	I12.0,I13.11,I13.2,N18.4,N18.5,N18.6,Z49.01,Z49.02,Z49.31,Z49.32,Z91.15,Z94.0,Z99.2
Liver disease (mild)	A51.45,A52.74,B18.0,B18.1,B18.2,B18.8,B18.9,B19.10,B19.20,B19.9,B25.1,B58.1,K70.0,K70.10,K70.11,K70.2,K70.30,K70.31,K70.9,K71.3,K71.4,K71.50,K71.51,K71.6,K71.7,K71.8,K73.0,K73.1,K73.2,K73.8,K73.9,K74.0,K74.00,K74.01,K74.02,K74.1,K74.2,K74.3,K74.4,K74.5,K74.60,K74.69,K75.1,K75.2,K75.3,K75.4,K75.81,K75.89,K75.9,K76.0,K76.1,K76.2,K76.3,K76.4,K76.81,K76.89,K76.9,K77.X,702.2,702.3,703.2,703.3,704.4,705.4,456.0,456.1,456.20,456.21,571.0,571.2,571.3,571.40,571.41,571.42,571.43,571.44,571.45,571.46,571.47,571.48,571.49,571.5,571.6,571.8,571.9,572.3,572.8,573.5,V42.7
Liver disease (moderate or severe)	B18.0,B18.1,B18.2,B18.8,B18.9,B19.0,B19.11,B19.21,B25.1,B58.1,I85.00,I85.01,I85.11,I86.4,K70.40,K70.41,K72.10,K72.11,K72.90,K76.5,K76.6,K76.7
Peptic ulcer disease (excluding bleeding)	K25.0,K25.1,K25.2,K25.3,K25.4,K25.5,K25.6,K25.7,K25.9,K26.0,K26.1,K26.2,K26.3,K26.4,K26.5,K26.6,K26.7,K26.9,K27.0,K27.1,K27.2,K27.3,K27.4,K27.5,K27.6,K27.7,K27.9,K28.0,K28.1,K28.2,K28.3,K28.4,K28.5,K28.6,K28.7,K28.9,531.41,531.51,531.61,531.70,531.71,531.91,532.41,532.51,532.61,532.70,532.71,532.91,533.41,533.51,533.61,533.70,533.71,533.91,534.41,534.51,534.61,534.70,534.71,534.91
AIDS/HIV	B20.X,O98.711,O98.712,O98.713,O98.719,O98.72,O98.73,Z21.X,042.X,042.1,042.2,042.3,042.4,042.5,042.6,042.7,042.8,042.9,043.0,043.1,043.2,043.3,043.4,043.5,043.6,043.7,043.8,043.9,044.0,044.1,044.2,044.3,044.4,044.5,044.6,044.7,044.8,044.9,043.X,044.X
Lymphoma	C81.00,C81.01,C81.02,C81.03,C81.04,C81.05,C81.06,C81.07,C81.08,C81.09,C81.10,C81.11,C81.12,C81.13,C81.14,C81.15,C81.16,C81.17,C81.18,C81.19,C81.20,C81.21,C81.22,C81.23,C81.24,C81.25,C81.26,C81.27,C81.28,C81.29,C81.30,C81.31,C81.32,C81.33,C81.34,C81.35,C81.36,C81.37,C81.38,C81.39,C81.40,C81.41,C81.42,C81.43,C81.44,C81.45,C81.46,C81.47,C81.48,C81.49,C81.70,C81.71,C81.72,C81.73,C81.74,C81.75,C81.76,C81.77,C81.78,C81.79,C81.90,C81.91,C81.92,C81.93,C81.94,C81.95,C81.96,C81.97,C81.98,C81.99,C82.00,C82.01,C82.02,C82.03,C82.04,C82.05,C82.06,C82.07,C82.08,C82.09,C82.10,C82.11,C82.12,C82.13,C82.14,C82.15,C82.16,C82.17,C82.18,C82.19,C82.20,C82.21,C82.22,C82.23,C82.24,C82.25,C82.26,C82.27,C82.28,C82.29,C82.30,C82.31,C82.32,C82.33,C82.34,C82.35,C82.36,C82.37,C82.38,C82.39,C82.40,C82.41,C82.42,C82.43,C82.44,C82.45,C82.46,C82.47,C82.48,C82.49,C82.50,C82.51,C82.52,C82.53,C82.54,C82.55,C82.56,C82.57,C82.58,C82.59,C82.60,C82.61,C82.62,C82.63,C82.64,C82.65,C82.66,C82.67,C82.68,C82.69,C82.80,C82.81,C82.82,C82.83,C82.84,C82.85,C82.86,C82.87,C82.88,C82.89,C82.90,C82.91,C82.92,C82.93,C82.94,C82.95,C82.96,C82.97,C82.98,C82.99,C83.00,C83.01,C83.02,C83.03,C83.04,C83.05,C83.06,C83.07,C83.08,C83.09,C83.10,C83.11,C83.12,C83.13,C83.14,C83.15,C83.16,C83.17,C83.18,C83.19,C83.30,C83.31,C83.32,C83.33,C83.34,C83.35,C83.36,C83.37,C83.38,C83.39,C83.50,C83.51,C83.52,C83.53,C83.54,C83.55,C83.56,C83.57,C83.58,C83.59,C83.70,C83.71,C83.72,C83.73,C83.74,C83.75,C83.76,C83.77,C83.78,C83.79,C83.80,C83.81,C83.82,C83.83,C83.84,C83.85,C83.86,C83.87,C83.88,C83.89,C83.90,C83.91,C83.92,C83.93,C83.94,C83.95,C83.96,C83.97,C83.98,C83.99,C84.00,C84.01,C84.02,C84.03,C84.04,C84.05,C84.06,C84.07,C84.08,C84.09,C84.10,C84.11,C84.12,C84.13,C84.14,C84.15,C84.16,C84.17,C84.18,C84.19,C84.40,C84.41,C84.42,C84.43,C84.44,C84.45,C84.46,C84.47,C84.48,C84.49,C84.60,C84.61,C84.62,C84.63,C84.64,C84.65,C84.66,C84.67,C84.68,C84.69,C84.70,C84.71,C84.72,C84.73,C84.74,C84.75,C84.76,C84.77,C84.78,C84.79,C84.90,C84.91,C84.92,C84.93,C84.94,C84.95,C84.96,C84.97,C84.98,C84.99,C84.A0,C84.A1,C84.A2,C84.A3,C84.A4,C84.A5,C84.A6,C84.A7,C84.A8,C84.A9,C84.Z0,C84.Z1,C84.Z2,C84.Z3,C84.Z4,C84.Z5,C84.Z6,C84.Z7,C84.Z8,C84.Z9,C85.10,C85.11,C85.12,C85.13,C85.14,C85.15,C85.16,C85.17,C85.18,C85.19,C85.20,C85.21,C85.22,C85.23,C85.24,C85.25,C85.26,C85.27,C85.28,C85.29,C85.80,C85.81,C85.82,C85.83,C85.84,C85.85,C85.86,C85.87,C85.88,C85.89,C85.90,C85.91,C85.92,C85.93,C85.94,C85.95,C85.96,C85.97,C85.98,C85.99,C86.0,C86.1,C86.2,C86.3,C86.4,C86.5,C86.6,C88.0,C88.2,C88.3,C88.4,C88.8,C88.9,C90.00,C90.01,C90.02,C90.20,C90.21,C90.22,C90.30,C90.31,C90.32,C96.0,C96.2,C96.20,C96.21,C96.22,C96.29,C96.4,C96.9,C96.A,C96.Z,D47.Z9,200.00,200.01,200.02,200.03,200.04,200.05,200.06,200.07,200.08,200.09,200.10,200.11,200.12,200.13,200.14,200.15,

© 2023 Essien UR et al. *JAMA Network Open*.

Condition	ICD-9-CM, ICD-10-CM, and CPT Codes
Lymphoma (*continued*)	200.16,200.17,200.18,200.19,200.20,200.21,200.22,200.23,200.24,200.25,200.26,200.27,200.28,200.29,200.30,200.31,200.32,200.33,200.34,200.35,200.36,200.37,200.38,200.39,200.40,200.41,200.42,200.43,200.44,200.45,200.46,200.47,200.48,200.49,200.50,200.51,200.52,200.53,200.54,200.55,200.56,200.57,200.58,200.59,200.60,200.61,200.62,200.63,200.64,200.65,200.66,200.67,200.68,200.69,200.70,200.71,200.72,200.73,200.74,200.75,200.76,200.77,200.78,200.79,200.80,200.81,200.82,200.83,200.84,200.85,200.86,200.87,200.88,200.89,200.90,200.91,200.92,200.93,200.94,200.95,200.96,200.97,200.98,200.99,201.00,201.01,201.02,201.03,201.04,201.05,201.06,201.07,201.08,201.09,201.10,201.11,201.12,201.13,201.14,201.15,201.16,201.17,201.18,201.19,201.20,201.21,201.22,201.23,201.24,201.25,201.26,201.27,201.28,201.29,201.30,201.31,201.32,201.33,201.34,201.35,201.36,201.37,201.38,201.39,201.40,201.41,201.42,201.43,201.44,201.45,201.46,201.47,201.48,201.49,201.50,201.51,201.52,201.53,201.54,201.55,201.56,201.57,201.58,201.59,201.60,201.61,201.62,201.63,201.64,201.65,201.66,201.67,201.68,201.69,201.70,201.71,201.72,201.73,201.74,201.75,201.76,201.77,201.78,201.79,201.80,201.81,201.82,201.83,201.84,201.85,201.86,201.87,201.88,201.89,201.90,201.91,201.92,201.93,201.94,201.95,201.96,201.97,201.98,201.99,202.00,202.01,202.02,202.03,202.04,202.05,202.06,202.07,202.08,202.09,202.10,202.11,202.12,202.13,202.14,202.15,202.16,202.17,202.18,202.19,202.20,202.21,202.22,202.23,202.24,202.25,202.26,202.27,202.28,202.29,202.30,202.31,202.32,202.33,202.34,202.35,202.36,202.37,202.38,202.50,202.51,202.52,202.53,202.54,202.55,202.56,202.57,202.58,202.59,202.60,202.61,202.62,202.63,202.64,202.65,202.66,202.67,202.68,202.69,202.70,202.71,202.72,202.73,202.74,202.75,202.76,202.77,202.78,202.79,202.80,202.81,202.82,202.83,202.84,202.85,202.86,202.87,202.88,202.89,202.90,202.91,202.92,202.93,202.94,202.95,202.96,202.97,202.98,202.99,203.00,203.01,203.02,203.03,203.04,203.05,203.06,203.07,203.08,203.09,203.10,203.11,203.12,203.13,203.14,203.15,203.16,203.17,203.18,203.19,203.20,203.21,203.22,203.23,203.24,203.25,203.26,203.27,203.28,203.29,203.30,203.31,203.32,203.33,203.34,203.35,203.36,203.37,203.38,203.39,203.40,203.41,203.42,203.43,203.44,203.45,203.46,203.47,203.48,203.49,203.50,203.51,203.52,203.53,203.54,203.55,203.56,203.57,203.58,203.59,203.60,203.61,203.62,203.63,203.64,203.65,203.66,203.67,203.68,203.69,203.70,203.71,203.72,203.73,203.74,203.75,203.76,203.77,203.78,203.79,203.80,203.81,238.6,273.3
Leukemia	C90.10,C90.11,C90.12,C91.00,C91.01,C91.02,C91.10,C91.11,C91.12,C91.30,C91.31,C91.32,C91.40,C91.41,C91.42,C91.50,C91.51,C91.52,C91.60,C91.61,C91.62,C91.90,C91.91,C91.92,C91.A0,C91.A1,C91.A2,C91.Z0,C91.Z1,C91.Z2,C92.00,C92.01,C92.02,C92.10,C92.11,C92.12,C92.20,C92.21,C92.22,C92.30,C92.31,C92.32,C92.40,C92.41,C92.42,C92.50,C92.51,C92.52,C92.60,C92.61,C92.62,C92.90,C92.91,C92.92,C92.A0,C92.A1,C92.A2,C92.Z0,C92.Z1,C92.Z2,C93.00,C93.01,C93.02,C93.10,C93.11,C93.12,C93.30,C93.31,C93.32,C93.90,C93.91,C93.92,C93.Z0,C93.Z1,C93.Z2,C94.00,C94.01,C94.02,C94.20,C94.21,C94.22,C94.30,C94.31,C94.32,C94.40,C94.41,C94.42,C94.6,C94.80,C94.81,C94.82,C95.00,C95.01,C95.02,C95.10,C95.11,C95.12,C95.90,C95.91,C95.92
Cancer (in situ)	D00.00,D00.01,D00.02,D00.03,D00.04,D00.05,D00.06,D00.07,D00.08,D00.1,D00.2,D01.0,D01.1,D01.2,D01.3,D01.40,D01.49,D01.5,D01.7,D01.9,D02.0,D02.1,D02.20,D02.21,D02.22,D02.3,D02.4,D03.0,D03.10,D03.11,D03.111,D03.112,D03.12,D03.121,D03.122,D03.20,D03.21,D03.22,D03.30,D03.39,D03.4,D03.51,D03.52,D03.59,D03.60,D03.61,D03.62,D03.70,D03.71,D03.72,D03.8,D03.9,D04.0,D04.10,D04.11,D04.111,D04.112,D04.12,D04.121,D04.122,D04.20,D04.21,D04.22,D04.30,D04.39,D04.4,D04.5,D04.60,D04.61,D04.62,D04.70,D04.71,D04.72,D04.8,D04.9,D05.00,D05.01,D05.02,D05.10,D05.11,D05.12,D05.80,D05.81,D05.82,D05.90,D05.91,D05.92,D06.0,D06.1,D06.7,D06.9,D07.0,D07.1,D07.2,D07.30,D07.39,D07.4,D07.5,D07.60,D07.61,D07.69,D09.0,D09.10,D09.19,D09.20,D09.21,D09.22,D09.3,D09.8,D09.9,140.0,140.1,140.2,140.3,140.4,140.5,140.6,140.7,140.8,140.9,141.0,141.1,141.2,141.3,141.4,141.5,141.6,141.7,141.8,141.9,142.0,142.1,142.2,142.3,142.4,142.5,142.6,142.7,142.8,142.9,143.0,143.1,143.2,143.3,143.4,143.5,143.6,143.7,143.8,143.9,144.0,144.1,144.2,144.3,144.4,144.5,144.6,144.7,144.8,144.9,145.0,145.1,145.2,145.3,145.4,145.5,145.6,145.7,145.8,145.9,146.0,146.1,146.2,146.3,146.4,146.5,146.6,146.7,146.8,146.9,147.0,147.1,147.2,147.3,147.4,147.5,147.6,147.7,147.8,147.9,148.0,148.1,148.2,148.3,148.4,148.5,148.6,148.7,148.8,148.9,149.0,149.1,149.2,149.3,149.4,149.5,149.6,149.7,149.8,149.9,150.0,150.1,150.2,150.3,150.4,150.5,150.6,150.7,150.8,150.9,151.0,151.1,151.2,151.3,151.4,151.5,151.6,151.7,151.8,151.9,152.0,152.1,152.2,152.3,152.4,152.5,152.6,152.7,152.8,152.9,153.0,153.1,153.2,153.3,153.4,153.5,153.6,153.7,153.8,153.9,154.0,154.1,154.2,154.3,154.4,154.5,154.6,154.7,154.8,154.9,155.0,155.1,155.2,155.3,155.4,155.5,155.6,155.7,155.8,155.9,156.0,156.1,156.2,156.3,156.4,156.5,156.6,156.7,156.8,156.9,157.0,157.1,157.2,157.3,157.4,157.5,157.6,157.7,157.8,157.9,158.0,158.1,158.2,158.3,158.4,158.5,158.6,158.7,158.8,158.9,159.0,159.1,159.2,159.3,159.4,159.5,159.6,159.7,159.8,159.9,160.0,160.1,160.2,160.3,160.4,160.5,160.6,160.7,160.8,160.9,161.0,161.1,161.2,161.3,161.4,

© 2023 Essien UR et al. *JAMA Network Open*.

Condition	ICD-9-CM, ICD-10-CM, and CPT Codes
Cancer (in situ, *continued*)	161.5,161.6,161.7,161.8,161.9,162.0,162.1,162.2,162.3,162.4,162.5,162.6,162.7,162.8,162.9,163.0,163.1,163.2,163.3,163.4,163.5,163.6,163.7,163.8,163.9,164.0,164.1,164.2,164.3,164.4,164.5,164.6,164.7,164.8,164.9,165.0,165.1,165.2,165.3,165.4,165.5,165.6,165.7,165.8,165.9,166.0,166.1,166.2,166.3,166.4,166.5,166.6,166.7,166.8,166.9,167.0,167.1,167.2,167.3,167.4,167.5,167.6,167.7,167.8,167.9,168.0,168.1,168.2,168.3,168.4,168.5,168.6,168.7,168.8,168.9,169.0,169.1,169.2,169.3,169.4,169.5,169.6,169.7,169.8,169.9,170.0,170.1,170.2,170.3,170.4,170.5,170.6,170.7,170.8,170.9,171.0,171.1,171.2,171.3,171.4,171.5,171.6,171.7,171.8,171.9,172.0,172.1,172.2,172.3,172.4,172.5,172.6,172.7,172.8,172.9,174.0,174.1,174.2,174.3,174.4,174.5,174.6,174.7,174.8,174.9,175.0,175.1,175.2,175.3,175.4,175.5,175.6,175.7,175.8,175.9,179.0,179.1,179.2,179.3,179.4,179.5,179.6,179.7,179.8,179.9,180.0,180.1,180.2,180.3,180.4,180.5,180.6,180.7,180.8,180.9,181.0,181.1,181.2,181.3,181.4,181.5,181.6,181.7,181.8,181.9,182.0,182.1,182.2,182.3,182.4,182.5,182.6,182.7,182.8,182.9,183.0,183.1,183.2,183.3,183.4,183.5,183.6,183.7,183.8,183.9,184.0,184.1,184.2,184.3,184.4,184.5,184.6,184.7,184.8,184.9,185.0,185.1,185.2,185.3,185.4,185.5,185.6,185.7,185.8,185.9,186.0,186.1,186.2,186.3,186.4,186.5,186.6,186.7,186.8,186.9,187.0,187.1,187.2,187.3,187.4,187.5,187.6,187.7,187.8,187.9,188.0,188.1,188.2,188.3,188.4,188.5,188.6,188.7,188.8,188.9,189.0,189.1,189.2,189.3,189.4,189.5,189.6,189.7,189.8,189.9,190.0,190.1,190.2,190.3,190.4,190.5,190.6,190.7,190.8,190.9,191.0,191.1,191.2,191.3,191.4,191.5,191.6,191.7,191.8,191.9,192.0,192.1,192.2,192.3,192.4,192.5,192.6,192.7,192.8,192.9,193.0,193.1,193.2,193.3,193.4,193.5,193.6,193.7,193.8,193.9,194.0,194.1,194.2,194.3,194.4,194.5,194.6,194.7,194.8,194.9,195.0,195.1,195.2,195.3,195.4,195.5,195.6,195.7,195.8,209.00,209.01,209.02,209.03,209.04,209.05,209.06,209.07,209.08,209.09,209.10,209.11,209.12,209.13,209.14,209.15,209.16,209.17,209.18,209.19,209.20,209.21,209.22,209.23,209.24,209.25,209.26,209.27,209.28,209.29,209.3,209.30,209.31,209.32,209.33,209.34,209.35,209.36,258.01,258.02,258.03
Metastatic cancer	C77.0,C77.1,C77.2,C77.3,C77.4,C77.5,C77.8,C77.9,C78.00,C78.01,C78.02,C78.1,C78.2,C78.30,C78.39,C78.4,C78.5,C78.6,C78.7,C78.80,C78.89,C79.00,C79.01,C79.02,C79.10,C79.11,C79.19,C79.2,C79.31,C79.32,C79.40,C79.49,C79.51,C79.52,C79.60,C79.61,C79.62,C79.70,C79.71,C79.72,C79.81,C79.82,C79.89,C79.9,C7B.00,C7B.01,C7B.02,C7B.03,C7B.04,C7B.09,C7B.1,C7B.8,C80.0,196.0,196.1,196.2,196.3,196.4,196.5,196.6,196.7,196.8,196.9,197.0,197.1,197.2,197.3,197.4,197.5,197.6,197.7,197.8,197.9,198.0,198.1,198.2,198.3,198.4,198.5,198.6,198.7,198.8,198.9,199.0,199.1,209.70,209.71,209.72,209.73,209.74,209.75,209.79,789.51
Solid tumor without metastasis (malignant)	C00.0,C00.1,C00.2,C00.3,C00.4,C00.5,C00.6,C00.8,C00.9,C01.X,C02.0,C02.1,C02.2,C02.3,C02.4,C02.8,C02.9,C03.0,C03.1,C03.9,C04.0,C04.1,C04.8,C04.9,C05.0,C05.1,C05.2,C05.8,C05.9,C06.0,C06.1,C06.2,C06.80,C06.89,C06.9,C07.X,C08.0,C08.1,C08.9,C09.0,C09.1,C09.8,C09.9,C10.0,C10.1,C10.2,C10.3,C10.4,C10.8,C10.9,C11.0,C11.1,C11.2,C11.3,C11.8,C11.9,C12.X,C13.0,C13.1,C13.2,C13.8,C13.9,C14.0,C14.2,C14.8,C15.3,C15.4,C15.5,C15.8,C15.9,C16.0,C16.1,C16.2,C16.3,C16.4,C16.5,C16.6,C16.8,C16.9,C17.0,C17.1,C17.2,C17.3,C17.8,C17.9,C18.0,C18.1,C18.2,C18.3,C18.4,C18.5,C18.6,C18.7,C18.8,C18.9,C19.X,C20.X,C21.0,C21.1,C21.2,C21.8,C22.0,C22.1,C22.2,C22.3,C22.4,C22.7,C22.8,C22.9,C23.X,C24.0,C24.1,C24.8,C24.9,C25.0,C25.1,C25.2,C25.3,C25.4,C25.7,C25.8,C25.9,C26.0,C26.1,C26.9,C30.0,C30.1,C31.0,C31.1,C31.2,C31.3,C31.8,C31.9,C32.0,C32.1,C32.2,C32.3,C32.8,C32.9,C33.X,C34.00,C34.01,C34.02,C34.10,C34.11,C34.12,C34.2,C34.30,C34.31,C34.32,C34.80,C34.81,C34.82,C34.90,C34.91,C34.92,C37.X,C38.0,C38.1,C38.2,C38.3,C38.4,C38.8,C39.0,C39.9,C40.00,C40.01,C40.02,C40.10,C40.11,C40.12,C40.20,C40.21,C40.22,C40.30,C40.31,C40.32,C40.80,C40.81,C40.82,C40.90,C40.91,C40.92,C41.0,C41.1,C41.2,C41.3,C41.4,C41.9,C43.0,C43.10,C43.11,C43.111,C43.112,C43.12,C43.121,C43.122,C43.20,C43.21,C43.22,C43.30,C43.31,C43.39,C43.4,C43.51,C43.52,C43.59,C43.60,C43.61,C43.62,C43.70,C43.71,C43.72,C43.8,C43.9,C44.00,C44.09,C44.101,C44.102,C44.1021,C44.1022,C44.109,C44.1091,C44.1092,C44.131,C44.1321,C44.1322,C44.1391,C44.1392,C44.191,C44.192,C44.1921,C44.1922,C44.199,C44.1991,C44.1992,C44.201,C44.202,C44.209,C44.291,C44.292,C44.299,C44.300,C44.301,C44.309,C44.390,C44.391,C44.399,C44.40,C44.49,C44.500,C44.501,C44.509,C44.590,C44.591,C44.599,C44.601,C44.602,C44.609,C44.691,C44.692,C44.699,C44.701,C44.702,C44.709,C44.791,C44.792,C44.799,C44.80,C44.89,C44.90,C44.99,C45.0,C45.1,C45.2,C45.7,C45.9,C46.0,C46.1,C46.2,C46.3,C46.4,C46.50,C46.51,C46.52,C46.7,C46.9,C47.0,C47.10,C47.11,C47.12,C47.20,C47.21,C47.22,C47.3,C47.4,C47.5,C47.6,C47.8,C47.9,C48.0,C48.1,C48.2,C48.8,C49.0,C49.10,C49.11,C49.12,C49.20,C49.21,C49.22,C49.3,C49.4,C49.5,C49.6,C49.8,C49.9,C49.A0,C49.A1,C49.A2,C49.A3,C49.A4,C49.A5,C49.A9,C4A.0,C4A.10,C4A.11,C4A.111,C4A.112,C4A.12,C4A.121,C4A.122,C4A.20,C4A.21,C4A.22,C4A.30,C4A.31,C4A.39,C4A.4,C4A.51,C4A.52,C4A.59,C4A.60,C4A.61,C4A.62,C4A.70,C4A.71,C4A.72,C4A.8,C4A.9,C50.011,C50.012,C50.019,C50.021,C50.022,C50.029,C50.111,C50.112,C50.119,C50.121,C50.122,C50.129,C50.211,C50.212,C50.219,C50.221,C50.222,C50.229,C50.311,C50.312,C50.319,C50.321,C50.322,C50.329,

© 2023 Essien UR et al. *JAMA Network Open*.

Condition	ICD-9-CM, ICD-10-CM, and CPT Codes
Solid tumor without metastasis (malignant, *continued*)	C50.411,C50.412,C50.419,C50.421,C50.422,C50.429,C50.511,C50.512,C50.519,C50.521,C50.522,C50.529,C50.611,C50.612,C50.619,C50.621,C50.622,C50.629,C50.811,C50.812,C50.819,C50.821,C50.822,C50.829,C50.911,C50.912,C50.919,C50.921,C50.922,C50.929,C51.0,C51.1,C51.2,C51.8,C51.9,C52.X,C53.0,C53.1,C53.8,C53.9,C54.0,C54.1,C54.2,C54.3,C54.8,C54.9,C55.X,C56.1,C56.2,C56.9,C57.00,C57.01,C57.02,C57.10,C57.11,C57.12,C57.20,C57.21,C57.22,C57.3,C57.4,C57.7,C57.8,C57.9,C58.X,C60.0,C60.1,C60.2,C60.8,C60.9,C61.X,C62.00,C62.01,C62.02,C62.10,C62.11,C62.12,C62.90,C62.91,C62.92,C63.00,C63.01,C63.02,C63.10,C63.11,C63.12,C63.2,C63.7,C63.8,C63.9,C64.1,C64.2,C64.9,C65.1,C65.2,C65.9,C66.1,C66.2,C66.9,C67.0,C67.1,C67.2,C67.3,C67.4,C67.5,C67.6,C67.7,C67.8,C67.9,C68.0,C68.1,C68.8,C68.9,C69.00,C69.01,C69.02,C69.10,C69.11,C69.12,C69.20,C69.21,C69.22,C69.30,C69.31,C69.32,C69.40,C69.41,C69.42,C69.50,C69.51,C69.52,C69.60,C69.61,C69.62,C69.80,C69.81,C69.82,C69.90,C69.91,C69.92,C70.0,C70.1,C70.9,C71.0,C71.1,C71.2,C71.3,C71.4,C71.5,C71.6,C71.7,C71.8,C71.9,C72.0,C72.1,C72.20,C72.21,C72.22,C72.30,C72.31,C72.32,C72.40,C72.41,C72.42,C72.50,C72.59,C72.9,C73.X,C74.00,C74.01,C74.02,C74.10,C74.11,C74.12,C74.90,C74.91,C74.92,C75.0,C75.1,C75.2,C75.3,C75.4,C75.5,C75.8,C75.9,C76.0,C76.1,C76.2,C76.3,C76.40,C76.41,C76.42,C76.50,C76.51,C76.52,C76.8,C7A.00,C7A.010,C7A.011,C7A.012,C7A.019,C7A.020,C7A.021,C7A.022,C7A.023,C7A.024,C7A.025,C7A.026,C7A.029,C7A.090,C7A.091,C7A.092,C7A.093,C7A.094,C7A.095,C7A.096,C7A.098,C7A.1,C7A.8,D46.9,E31.21,E31.22,E31.23
Arthropathies	L40.50,L40.51,L40.54,L40.59,L90.0,L94.0,L94.1,L94.3,M01.X0,M01.X11,M01.X12,M01.X19,M01.X21,M01.X22,M01.X29,M01.X31,M01.X32,M01.X39,M01.X41,M01.X42,M01.X49,M01.X51,M01.X52,M01.X59,M01.X61,M01.X62,M01.X69,M01.X71,M01.X72,M01.X79,M01.X8,M01.X9,M02.00,M02.011,M02.012,M02.019,M02.021,M02.022,M02.029,M02.031,M02.032,M02.039,M02.041,M02.042,M02.049,M02.051,M02.052,M02.059,M02.061,M02.062,M02.069,M02.071,M02.072,M02.079,M02.08,M02.09,M02.10,M02.111,M02.112,M02.119,M02.121,M02.122,M02.129,M02.131,M02.132,M02.139,M02.141,M02.142,M02.149,M02.151,M02.152,M02.159,M02.161,M02.162,M02.169,M02.171,M02.172,M02.179,M02.18,M02.19,M02.20,M02.211,M02.212,M02.219,M02.221,M02.222,M02.229,M02.231,M02.232,M02.239,M02.241,M02.242,M02.249,M02.251,M02.252,M02.259,M02.261,M02.262,M02.269,M02.271,M02.272,M02.279,M02.28,M02.29,M02.30,M02.311,M02.312,M02.319,M02.321,M02.322,M02.329,M02.331,M02.332,M02.339,M02.341,M02.342,M02.349,M02.351,M02.352,M02.359,M02.361,M02.362,M02.369,M02.371,M02.372,M02.379,M02.38,M02.39,M02.80,M02.811,M02.812,M02.819,M02.821,M02.822,M02.829,M02.831,M02.832,M02.839,M02.841,M02.842,M02.849,M02.851,M02.852,M02.859,M02.861,M02.862,M02.869,M02.871,M02.872,M02.879,M02.88,M02.89,M02.9,M05.00,M05.011,M05.012,M05.019,M05.021,M05.022,M05.029,M05.031,M05.032,M05.039,M05.041,M05.042,M05.049,M05.051,M05.052,M05.059,M05.061,M05.062,M05.069,M05.071,M05.072,M05.079,M05.09,M05.10,M05.111,M05.112,M05.119,M05.121,M05.122,M05.129,M05.131,M05.132,M05.139,M05.141,M05.142,M05.149,M05.151,M05.152,M05.159,M05.161,M05.162,M05.169,M05.171,M05.172,M05.179,M05.19,M05.20,M05.211,M05.212,M05.219,M05.221,M05.222,M05.229,M05.231,M05.232,M05.239,M05.241,M05.242,M05.249,M05.251,M05.252,M05.259,M05.261,M05.262,M05.269,M05.271,M05.272,M05.279,M05.29,M05.30,M05.311,M05.312,M05.319,M05.321,M05.322,M05.329,M05.331,M05.332,M05.339,M05.341,M05.342,M05.349,M05.351,M05.352,M05.359,M05.361,M05.362,M05.369,M05.371,M05.372,M05.379,M05.39,M05.40,M05.411,M05.412,M05.419,M05.421,M05.422,M05.429,M05.431,M05.432,M05.439,M05.441,M05.442,M05.449,M05.451,M05.452,M05.459,M05.461,M05.462,M05.469,M05.471,M05.472,M05.479,M05.49,M05.50,M05.511,M05.512,M05.519,M05.521,M05.522,M05.529,M05.531,M05.532,M05.539,M05.541,M05.542,M05.549,M05.551,M05.552,M05.559,M05.561,M05.562,M05.569,M05.571,M05.572,M05.579,M05.59,M05.60,M05.611,M05.612,M05.619,M05.621,M05.622,M05.629,M05.631,M05.632,M05.639,M05.641,M05.642,M05.649,M05.651,M05.652,M05.659,M05.661,M05.662,M05.669,M05.671,M05.672,M05.679,M05.69,M05.70,M05.711,M05.712,M05.719,M05.721,M05.722,M05.729,M05.731,M05.732,M05.739,M05.741,M05.742,M05.749,M05.751,M05.752,M05.759,M05.761,M05.762,M05.769,M05.771,M05.772,M05.779,M05.79,M05.7A,M05.80,M05.811,M05.812,M05.819,M05.821,M05.822,M05.829,M05.831,M05.832,M05.839,M05.841,M05.842,M05.849,M05.851,M05.852,M05.859,M05.861,M05.862,M05.869,M05.871,M05.872,M05.879,M05.89,M05.8A,M05.9,M06.00,M06.011,M06.012,M06.019,M06.021,M06.022,M06.029,M06.031,M06.032,M06.039,M06.041,M06.042,M06.049,M06.051,M06.052,M06.059,M06.061,M06.062,M06.069,M06.071,M06.072,M06.079,M06.08,M06.09,M06.0A,M06.1,M06.20,M06.211,M06.212,M06.219,M06.221,M06.222,M06.229,M06.231,M06.232,M06.239,M06.241,M06.242,M06.249,M06.251,M06.252,M06.259,M06.261,M06.262,M06.269,M06.271,M06.272,M06.279,M06.28,M06.29,M06.30,M06.311,M06.312,M06.319,M06.321,M06.322,M06.329,M06.331,M06.332,M06.339,M06.341,M06.342,M06.349,M06.351,M06.352,M06.359,M06.361,M06.362,M06.369,

© 2023 Essien UR et al. *JAMA Network Open*.

Condition	ICD-9-CM, ICD-10-CM, and CPT Codes
Arthropathies (*continued*)	M06.371,M06.372,M06.379,M06.38,M06.39,M06.4,M06.80,M06.811,M06.812,M06.819,M06.821,M06.822,M06.829,M06.831,M06.832,M06.839,M06.841,M06.842,M06.849,M06.851,M06.852,M06.859,M06.861,M06.862,M06.869,M06.871,M06.872,M06.879,M06.88,M06.89,M06.8A,M06.9,M07.60,M07.611,M07.612,M07.619,M07.621,M07.622,M07.629,M07.631,M07.632,M07.639,M07.641,M07.642,M07.649,M07.651,M07.652,M07.659,M07.661,M07.662,M07.669,M07.671,M07.672,M07.679,M07.68,M07.69,M08.00,M08.011,M08.012,M08.019,M08.021,M08.022,M08.029,M08.031,M08.032,M08.039,M08.041,M08.042,M08.049,M08.051,M08.052,M08.059,M08.061,M08.062,M08.069,M08.071,M08.072,M08.079,M08.08,M08.09,M08.0A,M08.1,M08.20,M08.211,M08.212,M08.219,M08.221,M08.222,M08.229,M08.231,M08.232,M08.239,M08.241,M08.242,M08.249,M08.251,M08.252,M08.259,M08.261,M08.262,M08.269,M08.271,M08.272,M08.279,M08.28,M08.29,M08.2A,M08.3,M08.40,M08.411,M08.412,M08.419,M08.421,M08.422,M08.429,M08.431,M08.432,M08.439,M08.441,M08.442,M08.449,M08.451,M08.452,M08.459,M08.461,M08.462,M08.469,M08.471,M08.472,M08.479,M08.48,M08.4A,M08.80,M08.811,M08.812,M08.819,M08.821,M08.822,M08.829,M08.831,M08.832,M08.839,M08.841,M08.842,M08.849,M08.851,M08.852,M08.859,M08.861,M08.862,M08.869,M08.871,M08.872,M08.879,M08.88,M08.89,M08.90,M08.911,M08.912,M08.919,M08.921,M08.922,M08.929,M08.931,M08.932,M08.939,M08.941,M08.942,M08.949,M08.951,M08.952,M08.959,M08.961,M08.962,M08.969,M08.971,M08.972,M08.979,M08.98,M08.99,M08.9A,M12.00,M12.011,M12.012,M12.019,M12.021,M12.022,M12.029,M12.031,M12.032,M12.039,M12.041,M12.042,M12.049,M12.051,M12.052,M12.059,M12.061,M12.062,M12.069,M12.071,M12.072,M12.079,M12.08,M12.09,M30.0,M30.1,M30.2,M30.3,M30.8,M31.0,M31.1,M31.2,M31.30,M31.31,M31.4,M31.5,M31.6,M31.7,M31.8,M31.9,M32.0,M32.10,M32.12,M32.13,M32.14,M32.15,M32.19,M32.8,M32.9,M33.00,M33.01,M33.02,M33.03,M33.09,M33.10,M33.11,M33.12,M33.13,M33.19,M33.20,M33.21,M33.22,M33.29,M33.90,M33.91,M33.92,M33.93,M33.99,M34.0,M34.1,M34.2,M34.81,M34.82,M34.83,M34.89,M34.9,M35.00,M35.01,M35.02,M35.03,M35.04,M35.09,M35.1,M35.2,M35.3,M35.5,M35.6,M35.8,M35.9,M36.0,M36.1,M36.8,M45.0,M45.1,M45.2,M45.3,M45.4,M45.5,M45.6,M45.7,M45.8,M45.9,M46.00,M46.01,M46.02,M46.03,M46.04,M46.05,M46.06,M46.07,M46.08,M46.09,M46.1,M46.50,M46.51,M46.52,M46.53,M46.54,M46.55,M46.56,M46.57,M46.58,M46.59,M46.80,M46.81,M46.82,M46.83,M46.84,M46.85,M46.86,M46.87,M46.88,M46.89,M46.90,M46.91,M46.92,M46.93,M46.94,M46.95,M46.96,M46.97,M46.98,M46.99,M49.80,M49.81,M49.82,M49.83,M49.84,M49.85,M49.86,M49.87,M49.88,M49.89,701.0,710.0,710.1,710.2,710.3,710.4,710.5,710.6,710.7,710.8,710.9,714.0,714.1,714.2,714.3,714.4,714.5,714.6,714.7,714.8,714.9,720.0,720.1,720.2,720.3,720.4,720.5,720.6,720.7,720.8,720.9,725.X
Coagulopathy	D61.09,D61.1,D61.2,D61.3,D61.810,D61.811,D61.818,D61.82,D61.89,D61.9,D65.X,D66.X,D67.X,D68.0,D68.1,D68.2,D68.311,D68.312,D68.318,D68.32,D68.4,D68.8,D68.9,D69.1,D69.3,D69.41,D69.42,D69.49,D69.51,D69.59,D69.6,D69.8,D69.9,D75.82,O99.111,O99.112,O99.113,O99.119,O99.12,O99.13,286.0,286.1,286.2,286.3,286.4,286.5,286.6,286.7,286.8,286.9,287.1,287.3,287.4,287.5,289.84,649.30,649.31,649.32,649.33,649.34
Obesity	E66.01,E66.09,E66.1,E66.2,E66.8,E66.9,O99.210,O99.211,O99.212,O99.213,O99.214,O99.215,R93.9,Z68.30,Z68.31,Z68.32,Z68.33,Z68.34,Z68.35,Z68.36,Z68.37,Z68.38,Z68.39,Z68.41,Z68.42,Z68.43,Z68.44,Z68.45,Z68.54,278.0,278.00,278.01,278.03,649.10,649.11,649.12,649.13,649.14,793.91,V85.30,V85.31,V85.32,V85.33,V85.34,V85.35,V85.36,V85.37,V85.38,V85.39,V85.41,V85.42,V85.43,V85.44,V85.45,V85.54
Weight loss	E40.X,E41.X,E42.X,E43.X,E44.0,E44.1,E45.X,E46.X,E64.0,O25.10,O25.11,O25.12,O25.13,O25.2,O25.3,R63.4,R64.X,260.X,261.X,262.X,263.X,260.1,260.2,260.3,260.4,260.5,260.6,260.7,260.8,260.9,261.0,261.1,261.2,261.3,261.4,261.5,261.6,261.7,261.8,261.9,262.0,262.1,262.2,262.3,262.4,262.5,262.6,262.7,262.8,262.9,263.0,263.1,263.2,263.3,263.4,263.5,263.6,263.7,263.8,263.9,783.21,783.22
Blood loss anemia	D50.0,O90.81,O99.02,O99.03,280.0,648.20,648.21,648.22,648.23,648.24
Deficiency anemia	D50.1,D50.8,D50.9,D51.0,D51.1,D51.2,D51.3,D51.8,D51.9,D52.0,D52.1,D52.8,D52.9,D53.0,D53.1,D53.2,D53.8,D53.9,D63.0,D63.1,D63.8,D64.9,O99.011,O99.012,O99.013,O99.019,280.1,280.2,280.3,280.4,280.5,280.6,280.7,280.8,280.9,281.0,281.1,281.2,281.3,281.4,281.5,281.6,281.7,281.8,281.9,285.21,285.22,285.23,285.24,285.25,285.26,285.27,285.28,285.29,285.9
Alcohol abuse	F10.10,F10.11,F10.120,F10.121,F10.129,F10.130,F10.131,F10.132,F10.139,F10.14,F10.150,F10.151,F10.159,F10.180,F10.181,F10.182,F10.188,F10.19,F10.20,F10.21,F10.220,F10.221,F10.229,F10.230,F10.231,F10.232,F10.239,F10.24,F10.250,F10.251,F10.259,F10.26,F10.27,F10.280,F10.281,F10.282,F10.288,F10.29,F10.94,F10.950,F10.951,F10.959,F10.96,F10.97,F10.980,G62.1,I42.6,K29.20,K29.21,K70.10,K70.11,O99.310,O99.311,O99.312,O99.313,O99.314,O99.315,291.0,291.1,291.2,291.3,291.5,291.8,291.81,291.82,291.89,291.9,303.00,303.01,303.02,303.03,303.04,303.05,303.06,303.07,303.08,303.09,303.10,303.11,303.12,303.13,

© 2023 Essien UR et al. *JAMA Network Open*.

Condition	ICD-9-CM, ICD-10-CM, and CPT Codes
Alcohol abuse (*continued*)	303.14,303.15,303.16,303.17,303.18,303.19,303.20,303.21,303.22,303.23,303.24,303.25,303.26,303.27,303.28,303.29,303.30,303.31,303.32,303.33,303.34,303.35,303.36,303.37,303.38,303.39,303.40,303.41,303.42,303.43,303.44,303.45,303.46,303.47,303.48,303.49,303.50,303.51,303.52,303.53,303.54,303.55,303.56,303.57,303.58,303.59,303.60,303.61,303.62,303.63,303.64,303.65,303.66,303.67,303.68,303.69,303.70,303.71,303.72,303.73,303.74,303.75,303.76,303.77,303.78,303.79,303.80,303.81,303.82,303.83,303.84,303.85,303.86,303.87,303.88,303.89,303.90,303.91,303.92,303.93,305.00,305.01,305.02,305.03
Drug abuse	F11.10,F11.11,F11.120,F11.121,F11.122,F11.129,F11.13,F11.14,F11.150,F11.151,F11.159,F11.181,F11.182,F11.188,F11.19,F11.20,F11.21,F11.220,F11.221,F11.222,F11.229,F11.23,F11.24,F11.250,F11.251,F11.259,F11.281,F11.282,F11.288,F11.29,F12.10,F12.11,F12.120,F12.121,F12.122,F12.129,F12.13,F12.150,F12.151,F12.159,F12.180,F12.188,F12.19,F12.20,F12.21,F12.220,F12.221,F12.222,F12.229,F12.23,F12.250,F12.251,F12.259,F12.280,F12.288,F12.29,F13.10,F13.11,F13.120,F13.121,F13.129,F13.130,F13.131,F13.132,F13.139,F13.14,F13.150,F13.151,F13.159,F13.180,F13.181,F13.182,F13.188,F13.19,F13.20,F13.21,F13.220,F13.221,F13.229,F13.230,F13.231,F13.232,F13.239,F13.24,F13.250,F13.251,F13.259,F13.26,F13.27,F13.280,F13.281,F13.282,F13.288,F13.29,F14.10,F14.11,F14.120,F14.121,F14.122,F14.129,F14.13,F14.14,F14.150,F14.151,F14.159,F14.180,F14.181,F14.182,F14.188,F14.19,F14.20,F14.21,F14.220,F14.221,F14.222,F14.229,F14.23,F14.24,F14.250,F14.251,F14.259,F14.280,F14.281,F14.282,F14.288,F14.29,F15.10,F15.11,F15.120,F15.121,F15.122,F15.129,F15.13,F15.14,F15.150,F15.151,F15.159,F15.180,F15.181,F15.182,F15.188,F15.19,F15.20,F15.21,F15.220,F15.221,F15.222,F15.229,F15.23,F15.24,F15.250,F15.251,F15.259,F15.280,F15.281,F15.282,F15.288,F15.29,F16.10,F16.11,F16.120,F16.121,F16.122,F16.129,F16.14,F16.150,F16.151,F16.159,F16.180,F16.183,F16.188,F16.19,F16.20,F16.21,F16.220,F16.221,F16.229,F16.24,F16.250,F16.251,F16.259,F16.280,F16.283,F16.288,F16.29,F18.10,F18.11,F18.120,F18.121,F18.129,F18.14,F18.150,F18.151,F18.159,F18.17,F18.180,F18.188,F18.19,F18.20,F18.21,F18.220,F18.221,F18.229,F18.24,F18.250,F18.251,F18.259,F18.27,F18.280,F18.288,F18.29,F19.10,F19.11,F19.120,F19.121,F19.122,F19.129,F19.130,F19.131,F19.132,F19.139,F19.14,F19.150,F19.151,F19.159,F19.16,F19.17,F19.180,F19.181,F19.182,F19.188,F19.19,F19.20,F19.21,F19.220,F19.221,F19.222,F19.229,F19.230,F19.231,F19.232,F19.239,F19.24,F19.250,F19.251,F19.259,F19.26,F19.27,F19.280,F19.281,F19.282,F19.288,F19.29,O99.320,O99.321,O99.322,O99.323,O99.324,O99.325,292.0,292.82,292.89,292.9,304.00,304.01,304.02,304.03,304.04,304.05,304.06,304.07,304.08,304.09,304.10,304.11,304.12,304.13,304.14,304.15,304.16,304.17,304.18,304.19,304.20,304.21,304.22,304.23,304.24,304.25,304.26,304.27,304.28,304.29,304.30,304.31,304.32,304.33,304.34,304.35,304.36,304.37,304.38,304.39,304.40,304.41,304.42,304.43,304.44,304.45,304.46,304.47,304.48,304.49,304.50,304.51,304.52,304.53,304.54,304.55,304.56,304.57,304.58,304.59,304.60,304.61,304.62,304.63,304.64,304.65,304.66,304.67,304.68,304.69,304.70,304.71,304.72,304.73,304.74,304.75,304.76,304.77,304.78,304.79,304.80,304.81,304.82,304.83,304.84,304.85,304.86,304.87,304.88,304.89,304.90,304.91,304.92,304.93,305.20,305.21,305.22,305.23,305.24,305.25,305.26,305.27,305.28,305.29,305.30,305.31,305.32,305.33,305.34,305.35,305.36,305.37,305.38,305.39,305.40,305.41,305.42,305.43,305.44,305.45,305.46,305.47,305.48,305.49,305.50,305.51,305.52,305.53,305.54,305.55,305.56,305.57,305.58,305.59,305.60,305.61,305.62,305.63,305.64,305.65,305.66,305.67,305.68,305.69,305.70,305.71,305.72,305.73,305.74,305.75,305.76,305.77,305.78,305.79,305.80,305.81,305.82,305.83,305.84,305.85,305.86,305.87,305.88,305.89,305.90,305.91,305.92,305.93,648.30,648.31,648.32,648.33,648.34
Psychoses	F06.0,F06.1,F06.2,F06.30,F06.33,F11.150,F11.151,F11.159,F11.250,F11.251,F11.259,F11.950,F11.951,F11.959,F12.150,F12.151,F12.159,F12.250,F12.251,F12.259,F12.950,F12.951,F12.959,F13.150,F13.151,F13.159,F13.250,F13.251,F13.259,F13.950,F13.951,F13.959,F14.150,F14.151,F14.159,F14.250,F14.251,F14.259,F14.950,F14.951,F14.959,F15.150,F15.151,F15.159,F15.250,F15.251,F15.259,F15.950,F15.951,F15.959,F16.150,F16.151,F16.159,F16.250,F16.251,F16.259,F16.950,F16.951,F16.959,F18.150,F18.151,F18.159,F18.250,F18.251,F18.259,F18.950,F18.951,F18.959,F19.150,F19.151,F19.159,F19.250,F19.251,F19.259,F19.950,F19.951,F19.959,F20.0,F20.1,F20.2,F20.3,F20.5,F20.81,F20.89,F20.9,F21.X,F22.X,F23.X,F24.X,F25.0,F25.1,F25.8,F25.9,F28.X,F29.X,F30.10,F30.11,F30.12,F30.13,F30.2,F30.3,F30.4,F30.8,F30.9,F31.0,F31.10,F31.11,F31.12,F31.13,F31.2,F31.30,F31.31,F31.32,F31.4,F31.5,F31.60,F31.61,F31.62,F31.63,F31.64,F31.70,F31.71,F31.72,F31.73,F31.74,F31.75,F31.76,F31.77,F31.78,F31.81,F31.89,F31.9,F32.4,F32.5,F33.40,F33.41,F33.42,F34.0,F34.8,F34.81,F34.89,F34.9,F39.X,F44.89,F84.3,295.00,295.01,295.02,295.03,295.04,295.05,295.06,295.07,295.08,295.09,295.10,295.11,295.12,295.13,295.14,295.15,295.16,295.17,295.18,295.19,295.20,295.21,295.22,295.23,295.24,295.25,295.26,295.27,295.28,295.29,295.30,295.31,295.32,295.33,295.34,295.35,295.36,295.37,295.38,295.39,295.40,295.41,295.42,295.43,295.44,295.45,295.46,295.47,

© 2023 Essien UR et al. *JAMA Network Open*.

Condition	ICD-9-CM, ICD-10-CM, and CPT Codes
Psychoses (*continued*)	295.48,295.49,295.50,295.51,295.52,295.53,295.54,295.55,295.56,295.57,295.58,295.59,295.60,295.61,295.62,295.63,295.64,295.65,295.66,295.67,295.68,295.69,295.70,295.71,295.72,295.73,295.74,295.75,295.76,295.77,295.78,295.79,295.80,295.81,295.82,295.83,295.84,295.85,295.86,295.87,295.88,295.89,295.90,295.91,295.92,295.93,295.94,295.95,295.96,295.97,295.98,295.99,296.00,296.01,296.02,296.03,296.04,296.05,296.06,296.07,296.08,296.09,296.10,296.11,296.12,296.13,296.14,296.15,296.16,296.17,296.18,296.19,296.20,296.21,296.22,296.23,296.24,296.25,296.26,296.27,296.28,296.29,296.30,296.31,296.32,296.33,296.34,296.35,296.36,296.37,296.38,296.39,296.40,296.41,296.42,296.43,296.44,296.45,296.46,296.47,296.48,296.49,296.50,296.51,296.52,296.53,296.54,296.55,296.56,296.57,296.58,296.59,296.60,296.61,296.62,296.63,296.64,296.65,296.66,296.67,296.68,296.69,296.70,296.71,296.72,296.73,296.74,296.75,296.76,296.77,296.78,296.79,296.80,296.81,296.82,296.83,296.84,296.85,296.86,296.87,296.88,296.89,296.90,296.91,296.92,296.93,296.94,296.95,296.96,296.97,296.98,296.99,297.00,297.01,297.02,297.03,297.04,297.05,297.06,297.07,297.08,297.09,297.10,297.11,297.12,297.13,297.14,297.15,297.16,297.17,297.18,297.19,297.20,297.21,297.22,297.23,297.24,297.25,297.26,297.27,297.28,297.29,297.30,297.31,297.32,297.33,297.34,297.35,297.36,297.37,297.38,297.39,297.40,297.41,297.42,297.43,297.44,297.45,297.46,297.47,297.48,297.49,297.50,297.51,297.52,297.53,297.54,297.55,297.56,297.57,297.58,297.59,297.60,297.61,297.62,297.63,297.64,297.65,297.66,297.67,297.68,297.69,297.70,297.71,297.72,297.73,297.74,297.75,297.76,297.77,297.78,297.79,297.80,297.81,297.82,297.83,297.84,297.85,297.86,297.87,297.88,297.89,297.90,297.91,297.92,297.93,297.94,297.95,297.96,297.97,297.98,297.99,298.00,298.01,298.02,298.03,298.04,298.05,298.06,298.07,298.08,298.09,298.10,298.11,298.12,298.13,298.14,298.15,298.16,298.17,298.18,298.19,298.20,298.21,298.22,298.23,298.24,298.25,298.26,298.27,298.28,298.29,298.30,298.31,298.32,298.33,298.34,298.35,298.36,298.37,298.38,298.39,298.40,298.41,298.42,298.43,298.44,298.45,298.46,298.47,298.48,298.49,298.50,298.51,298.52,298.53,298.54,298.55,298.56,298.57,298.58,298.59,298.60,298.61,298.62,298.63,298.64,298.65,298.66,298.67,298.68,298.69,298.70,298.71,298.72,298.73,298.74,298.75,298.76,298.77,298.78,298.79,298.80,298.81,298.82,298.83,298.84,298.85,298.86,298.87,298.88,298.89,298.9,298.90,299.10,299.11
Depression	F06.31,F06.32,F06.34,F32.0,F32.1,F32.2,F32.3,F32.8,F32.81,F32.89,F32.9,F33.0,F33.1,F33.2,F33.3,F33.8,F33.9,F34.1,300.4,301.12,309.0,309.1,311.X
Cerebrovascular disease	G45.0,G45.1,G45.2,G45.3,G45.4,G45.8,G45.9,G46.0,G46.1,G46.2,G46.3,G46.4,G46.5,G46.6,G46.7,G46.8,H34.00,H34.01,H34.02,H34.03,H34.10,H34.11,H34.12,H34.13,H34.211,H34.212,H34.213,H34.219,H34.231,H34.232,H34.233,H34.239,I60.00,I60.01,I60.02,I60.10,I60.11,I60.12,I60.2,I60.20,I60.21,I60.22,I60.30,I60.31,I60.32,I60.4,I60.50,I60.51,I60.52,I60.6,I60.7,I60.8,I60.9,I61.0,I61.1,I61.2,I61.3,I61.4,I61.5,I61.6,I61.8,I61.9,I62.00,I62.03,I62.1,I62.9,I63.00,I63.011,I63.012,I63.013,I63.019,I63.02,I63.031,I63.032,I63.033,I63.039,I63.09,I63.10,I63.111,I63.112,I63.113,I63.119,I63.12,I63.131,I63.132,I63.133,I63.139,I63.19,I63.20,I63.211,I63.212,I63.213,I63.219,I63.22,I63.231,I63.232,I63.233,I63.239,I63.29,I63.30,I63.311,I63.312,I63.313,I63.319,I63.321,I63.322,I63.323,I63.329,I63.331,I63.332,I63.333,I63.339,I63.341,I63.342,I63.343,I63.349,I63.39,I63.40,I63.411,I63.412,I63.413,I63.419,I63.421,I63.422,I63.423,I63.429,I63.431,I63.432,I63.433,I63.439,I63.441,I63.442,I63.443,I63.449,I63.49,I63.50,I63.511,I63.512,I63.513,I63.519,I63.521,I63.522,I63.523,I63.529,I63.531,I63.532,I63.533,I63.539,I63.541,I63.542,I63.543,I63.549,I63.59,I63.6,I63.8,I63.81,I63.89,I63.9,I65.01,I65.02,I65.03,I65.09,I65.1,I65.21,I65.22,I65.23,I65.29,I65.8,I65.9,I66.01,I66.02,I66.03,I66.09,I66.11,I66.12,I66.13,I66.19,I66.21,I66.22,I66.23,I66.29,I66.3,I66.8,I66.9
Cerebrovascular disease sequelae	I69.30,I69.31,I69.310,I69.311,I69.312,I69.313,I69.314,I69.315,I69.318,I69.319,I69.320,I69.321,I69.322,I69.323,I69.328,I69.331,I69.332,I69.333,I69.334,I69.339,I69.341,I69.342,I69.343,I69.344,I69.349,I69.351,I69.352,I69.353,I69.354,I69.359,I69.361,I69.362,I69.363,I69.364,I69.365,I69.369,I69.390,I69.391,I69.392,I69.393,I69.398,I69.80,I69.81,I69.810,I69.811,I69.812,I69.813,I69.814,I69.815,I69.818,I69.819,I69.820,I69.821,I69.822,I69.823,I69.828,I69.831,I69.832,I69.833,I69.834,I69.839,I69.841,I69.842,I69.843,I69.844,I69.849,I69.851,I69.852,I69.853,I69.854,I69.859,I69.861,I69.862,I69.863,I69.864,I69.865,I69.869,I69.890,I69.891,I69.892,I69.893,I69.898,I69.90,I69.91,I69.910,I69.911,I69.912,I69.913,I69.914,I69.915,I69.918,I69.919,I69.920,I69.921,I69.922,I69.923,I69.928,I69.931,I69.932,I69.933,I69.934,I69.939,I69.941,I69.942,I69.943,I69.944,I69.949,I69.951,I69.952,I69.953,I69.954,I69.959,I69.961,I69.962,I69.963,I69.964,I69.965,I69.969,I69.990,I69.991,I69.992,I69.993,I69.998,P91.821,P91.822,P91.823,P91.829
Other thyroid disorders	E04.0,E04.1,E04.2,E04.8,E04.9,E05.00,E05.01,E05.10,E05.11,E05.20,E05.21,E05.30,E05.31,E05.40,E05.41,E05.80,E05.81,E05.90,E05.91,E06.0,E06.1,E06.2,E06.3,E06.4,E06.5,E06.9,O90.5
Dementia	F01.50,F01.51,F02.80,F02.81,F03.90,F03.91,G30.0,G30.1,G30.8,G30.9,G31.01,G31.09,G31.1,G31.2,G31.81,G31.82,G31.83,G31.85,G31.89,G31.9

© 2023 Essien UR et al. *JAMA Network Open*.

**eTable 2:** Characteristics of individuals using combination SGLT2i and GLP1-RA therapy			
	**Combination (n=4,389)**		
	**Low copayment (n=1,294)**	**Medium copayment (n=1,949)**	**High copayment (n=1,146)**
Age, mean (SD)	60.1 (10.5)	55.7 (10.5)	58.7 (12)
Female sex, n (%)	683 (52.8%)	764 (39.2%)	470 (41.0%)
**Race**			
Asian, n (%)	47 (3.6%)	64 (3.3%)	32 (2.8%)
Black, n (%)	237 (18.3%)	237 (12.2%)	137 (12.0%)
Hispanic, n (%)	275 (21.3%)	325 (16.7%)	213 (18.6%)
Unknown, n (%)	63 (4.9%)	51 (2.6%)	50 (4.4%)
White, n (%)	672 (51.9%)	1272 (65.3%)	714 (62.3%)
**Household income (USD $)**			
< $40,000, n (%)	575 (44.4%)	414 (21.2%)	270 (23.6%)
$40-<$50,000, n (%)	125 (9.7%)	159 (8.2%)	85 (7.4%)
$50-<$60,000, n (%)	108 (8.4%)	156 (8.0%)	105 (9.2%)
$60-<$75,000, n (%)	102 (7.9%)	212 (10.9%)	145 (12.7%)
$75-<$100,000, n (%)	120 (9.3%)	335 (17.2%)	183 (16.0%)
≥ $100,000, n (%)	140 (10.8%)	582 (29.9%)	290 (25.3%)
Unknown, n (%)	124 (9.6%)	91 (4.7%)	68 (5.9%)
**Education attainment**			
< 12th Grade, n (%)	20 (1.6%)	8 (0.4%)	7 (0.6%)
High school diploma, n (%)	567 (43.8%)	570 (29.3%)	332 (29.0%)
< Bachelor's degree, n (%)	578 (44.7%)	1056 (54.2%)	643 (56.1%)
Bachelor's degree +, n (%)	83 (6.4%)	291 (14.9%)	136 (11.9%)
Unknown, n (%)	46 (3.6%)	24 (1.2%)	28 (2.4%)
**Insurance type**			
Medicare beneficiary, n (%)	933 (72.1%)	318 (16.3%)	440 (38.4%)
Medicare (unspecified), n (%)	170 (13.1%)	310 (15.9%)	422 (36.8%)
Low-income subsidy, n (%)	510 (39.4%)	7 (0.4%)	9 (0.8%)
Dual Medicare/Medicaid, n (%)	253 (19.6%)	1 (0.1%)	9 (0.8%)
Enrollment months after index date, median (Q1, Q3)	27.8 (20.4, 39.8)	26.7 (18.5, 39)	28.2 (20.4, 40.3)
**Healthcare utilization**			
Cardiology encounters, median (Q1, Q3)	0 (0, 2)	0 (0, 1)	0 (0, 1)
Endocrinology encounters, median (Q1, Q3)	0 (0, 1)	0 (0, 2)	0 (0, 2)
PCP encounters, median (Q1, Q3)	20 (11, 34)	12 (7, 20)	12 (7, 20)
Any deductible contribution from medications, n (%)	359 (27.7%)	303 (15.6%)	217 (18.9%)
**Comorbidities**			
T2DM qualifying diagnosis, n (%)	1282 (99.1%)	1936 (99.3%)	1140 (99.5%)
HF qualifying diagnosis, n (%)	188 (14.5%)	107 (5.5%)	94 (8.2%)
Elixhauser comorbidities (Q1, Q3)	6 (5, 9)	5 (4, 7)	5 (4, 7)

© 2023 Essien UR et al. *JAMA Network Open*.

**eTable 3:** Adherence rates to GLP1-RA or SGLT2i therapy adjusted for sociodemographic factors and stratified by copayment							
**Glucagon-Like Peptide-1 Receptor Agonist**							
	**Low Copayment <$10/month**		**Medium Copayment $10-<$50/month**		**High Copayment ≥$50/month**		**Interaction**
	**OR (95% CI)**	**P**	**OR (95% CI)**	**P**	**OR (95% CI)**	**P**	**P-value**
**Educational attainment**							
<High school diploma	0.72 (0.48, 1.09)	0.12	0.67 (0.40, 1.10)	0.11	0.64 (0.38, 1.09)	0.10	0.488
High school graduate	1.09 (0.90, 1.32)	0.40	0.83 (0.71, 0.96)	0.01	0.94 (0.83, 1.07)	0.36	
Some college	1.18 (0.99, 1.42)	0.07	0.91 (0.80, 1.04)	0.17	0.98 (0.88, 1.09)	0.67	
≥Bachelor degree	Referent		Referent		Referent		
**Insurance type**							
Medicare—non-LIS/Dual	1.10 (0.94, 1.28)	0.23	0.85 (0.77, 0.93)	0.001	0.50 (0.46, 0.55)	<.0001	<.0001
Medicare—Low-income subsidy	1.06 (0.92, 1.21)	0.42	0.75 (0.52, 1.07)	0.11	0.47 (0.34, 0.66)	<.0001	
Dual Medicare/Medicaid	0.98 (0.84, 1.13)	0.73	0.63 (0.35, 1.11)	0.11	0.64 (0.42, 0.99)	0.04	
Commercial	Referent		Referent		Referent		
**Race and ethnicity**							
Asian	0.96 (0.72, 1.27)	0.76	0.71 (0.55, 0.91)	0.01	0.82 (0.67, 1.02)	0.07	0.366
Black	0.76 (0.69, 0.85)	<.0001	0.77 (0.68, 0.86)	<.0001	0.64 (0.58, 0.72)	<.0001	
Hispanic	0.88 (0.79, 0.99)	0.04	0.63 (0.56, 0.70)	<.0001	0.84 (0.76, 0.92)	0.0004	
White	Referent		Referent		Referent		
**Annual household income (USD $)**							
<$40,000	0.77 (0.64, 0.93)	0.008	0.68 (0.60, 0.77)	<.0001	0.72 (0.64, 0.80)	<.0001	0.987
$40-<$50,000	0.81 (0.64, 1.01)	0.06	0.83 (0.70, 0.98)	0.03	0.74 (0.64, 0.85)	<.0001	
$50-<$60,000	0.72 (0.57, 0.91)	0.006	0.76 (0.65, 0.89)	0.001	0.84 (0.73, 0.96)	0.009	
$60-<$75,000	0.85 (0.68, 1.07)	0.17	0.79 (0.68, 0.91)	0.001	0.81 (0.72, 0.91)	0.001	
$75-<$100,000	0.81 (0.65, 1.01)	0.06	0.92 (0.81, 1.05)	0.22	0.89 (0.80, 0.99)	0.03	
≥$100,000	Referent		Referent		Referent		
**Sex**							
Female	1.00 (0.91, 1.09)	0.93	0.83 (0.77, 0.90)	<.0001	0.87 (0.81, 0.93)	<.0001	0.006
Male	Referent		Referent		Referent		

© 2023 Essien UR et al. *JAMA Network Open*.

**eTable 3 continued: b)** Adherence rates to GLP1-RA or SGLT2i therapy adjusted for sociodemographic factors and stratified by copayment							
**Sodium-Glucose Cotransporter 2 Inhibitor**							
	**Low Copayment <$10/month**		**Medium Copayment $10-<$50/month**		**High Copayment ≥$50/month**		**Interaction**
	**OR (95% CI)**	**P**	**OR (95% CI)**	**P**	**OR (95% CI)**	**P**	**P-value**
**Educational attainment**							
<High school diploma	0.98 (0.67, 1.45)	0.92	0.68 (0.48, 0.95)	0.03	1.03 (0.62, 1.72)	0.90	0.518
High school graduate	0.87 (0.72, 1.06)	0.17	0.87 (0.77, 0.97)	0.01	0.87 (0.75, 1.01)	0.06	
Some college	0.94 (0.78, 1.13)	0.5	0.91 (0.83, 1.00)	0.06	0.90 (0.79, 1.02)	0.08	
≥Bachelor degree	Referent		Referent		Referent		
**Insurance type**							
Medicare—non-LIS/Dual	1.08 (0.93, 1.26)	0.33	0.46 (0.43, 0.50)	<.0001	0.72 (0.65, 0.79)	<.0001	0.009
Medicare—Low-income subsidy	1.11 (0.98, 1.26)	0.1	0.79 (0.59, 1.05)	0.11	0.73 (0.51, 1.05)	0.09	
Dual Medicare/Medicaid	1.20 (1.04, 1.38)	0.01	0.26 (0.15, 0.47)	<.0001	0.59 (0.35, 0.99)	0.05	
Commercial	Referent		Referent		Referent		
**Race and ethnicity**							
Asian	0.83 (0.68, 1.01)	0.06	0.70 (0.61, 0.80)	<.0001	0.76 (0.65, 0.90)	0.0012	0.0002
Black	0.61 (0.55, 0.68)	<.0001	0.57 (0.52, 0.62)	<.0001	0.66 (0.58, 0.74)	<.0001	
Hispanic	0.64 (0.57, 0.71)	<.0001	0.49 (0.45, 0.53)	<.0001	0.73 (0.65, 0.82)	<.0001	
White	Referent		Referent		Referent		
**Annual household income (USD $)**							
<$40,000	0.81 (0.68, 0.97)	0.02	0.67 (0.61, 0.74)	<.0001	0.64 (0.57, 0.73)	<.0001	0.501
$40-<$50,000	0.78 (0.64, 0.96)	0.02	0.71 (0.63, 0.80)	<.0001	0.71 (0.60, 0.83)	<.0001	
$50-<$60,000	0.86 (0.70, 1.07)	0.17	0.78 (0.69, 0.88)	<.0001	0.78 (0.67, 0.92)	0.0021	
$60-<$75,000	0.87 (0.70, 1.07)	0.17	0.92 (0.82, 1.02)	0.12	0.82 (0.71, 0.95)	0.010	
$75-<$100,000	0.99 (0.81, 1.21)	0.92	0.93 (0.84, 1.02)	0.11	0.97 (0.86, 1.10)	0.65	
≥$100,000	Referent		Referent		Referent		
**Sex**							
Female	0.95 (0.87, 1.04)	0.27	0.89 (0.83, 0.94)	0.0001	0.98 (0.90, 1.06)	0.55	0.014
Male	Referent		Referent		Referent		

© 2023 Essien UR et al. *JAMA Network Open*.

eTable continued: Adherence rates to GLP1-RA or SGLT2i therapy adjusted for sociodemographic factors and stratified by copayment

Combination Therapy							
	**Low Copayment <$10/month**		**Medium Copayment $10-<$50/month**		**High Copayment ≥$50/month**		**Interaction**
	**OR (95% CI)**	**P**	**OR (95% CI)**	**P**	**OR (95% CI)**	**P**	**P-value**
**Educational attainment**							
<High school diploma	1.89 (0.45, 7.93)	0.38	0.75 (0.16, 3.61)	0.72	1.25 (0.21, 7.33)	0.80	0.995
High school graduate	0.83 (0.43, 1.62)	0.59	0.97 (0.66, 1.44)	0.89	0.90 (0.54, 1.49)	0.67	
Some college	0.92 (0.49, 1.76)	0.81	1.07 (0.76, 1.51)	0.69	1.14 (0.72, 1.80)	0.57	
≥Bachelor degree	Referent		Referent		Referent		
**Insurance type**							
Medicare—non-LIS/Dual	1.20 (0.72, 2.00)	0.49	0.99 (0.70, 1.40)	0.97	0.76 (0.55, 1.04)	0.09	0.540
Medicare—Low-income subsidy	1.15 (0.77, 1.72)	0.49	0.32 (0.06, 1.59)	0.16	1.05 (0.25, 4.47)	0.95	
Dual Medicare/Medicaid	1.03 (0.64, 1.66)	0.89		0.97	0.71 (0.13, 3.92)	0.70	
Commercial	Referent		Referent		Referent		
**Race and ethnicity**							
Asian	0.70 (0.33, 1.49)	0.35	0.47 (0.27, 0.83)	0.01	2.89 (0.96, 8.71)	0.06	0.001
Black	0.67 (0.45, 0.97)	0.04	0.57 (0.41, 0.78)	0.0006	0.50 (0.33, 0.74)	0.0005	
Hispanic	0.61 (0.43, 0.88)	0.01	0.56 (0.42, 0.75)	<.0001	1.09 (0.76, 1.56)	0.65	
White	Referent		Referent		Referent		
**Annual household income**							
<$40,000	0.86 (0.48, 1.54)	0.62	0.76 (0.54, 1.07)	0.11	0.82 (0.54, 1.23)	0.33	0.157
$40-<$50,000	1.17 (0.57, 2.40)	0.66	0.63 (0.41, 0.98)	0.04	0.58 (0.33, 1.00)	0.05	
$50-<$60,000	0.99 (0.49, 2.03)	0.99	0.55 (0.36, 0.84)	0.01	0.81 (0.48, 1.36)	0.42	
$60-<$75,000	0.70 (0.36, 1.37)	0.30	0.64 (0.43, 0.94)	0.02	1.38 (0.84, 2.25)	0.21	
$75-<$100,000	1.17 (0.59, 2.31)	0.66	0.81 (0.57, 1.15)	0.23	0.97 (0.62, 1.49)	0.87	
≥$100,000	Referent		Referent		Referent		
**Sex**							
Female	0.94 (0.70, 1.25)	0.66	0.78 (0.63, 0.98)	0.03	0.92 (0.70, 1.20)	0.54	0.355
Male	Referent		Referent		Referent		

© 2023 Essien UR et al. *JAMA Network Open*.
