# Supplementary material for: Association of Prescription Co-payment With Adherence to Glucagon-Like Peptide-1 Receptor Agonist and Sodium-Glucose Cotransporter-2 Inhibitor Therapies in Patients With Heart Failure and Diabetes
Source: JAMA Netw Open. 2023 Jun 1;6(6):e2316290. doi: 10.1001/jamanetworkopen.2023.16290 (PMC10236237; doi:10.1001/jamanetworkopen.2023.16290)
Supplement: Supplement 2. — Data Sharing Statement [file jamanetwopen-e2316290-s002.pdf]

Data Sharing Statement

Essien. Association of Prescription Co-payment With Adherence to Glucagon-Like Peptide-1 Receptor Agonist and Sodium-Glucose Cotransporter-2 Inhibitor Therapies in Patients With Heart Failure and Diabetes. *JAMA Netw Open*. Published June 01, 2023.
doi:10.1001/jamanetworkopen.2023.16290

Data
Data available: No
